# Comparative mitogenome analysis reveals mitochondrial genome characteristics in eight strains of *Beauveria*

**DOI:** 10.7717/peerj.14067

**Published:** 2022-09-28

**Authors:** Yu Bai, Xuyuan Gao, Hui Wang, Lin Ye, Xianqun Zhang, Wei Huang, Xiuzhen Long, Kang Yang, Guoyong Li, Jianlin Luo, Jiyue Wang, Yonghao Yu

**Affiliations:** 1 College of Mathematics & Information Science, Guiyang University, Guiyang, China; 2 Guangxi Key Laboratory of Biology for Crop Diseases and Insect Pests, Guangxi Academy of Agricultural Sciences, Nanning, China; 3 Guizhou Provincial Key Laboratory for Rare Animal and Economic Insects of the Mountainous Region, Guiyang University, Guiyang, China; 4 College of Biology and Environmental Engineering, Guiyang University, Guiyang, China

**Keywords:** *Beauveria*, Mitochondrial genome, Mitogenome annotation, Comparative mitogenome, Illumina, Polycistronic transcript

## Abstract

Despite the significant progress that has been made in the genome sequencing of *Beauveria* species, mitochondrial genome (mitogenome) used to examine genetic diversity within fungal populations. Complete mitogenomes of *Beauveria* species can be easily sequenced and assembled using various sequencing techniques. However, since mitogenome annotations are mainly derived from similar species comparison and software prediction, and are not supported by RNA-seq transcripts data, it leads to problems with the accuracy of mitochondrial annotations and the inability to understand RNA processing. In this study, we assembled and annotated the mitogenome of eight *Beauveria* strains using Illumina DNA and RNA sequencing data. The circular mitogenome of eight *Beauveria* strains ranged from 26,850 bp (*B. caledonica* strain ATCC 64970) to 35,999 bp (*B. brongniartii* strain GYU-BMZ03), with the intronic insertions accounting for most of the size variation, thus contributing to a total mitochondrial genome (mitogenome) size of 7.01% and 28.95%, respectively. Intron number variations were not directly related to the evolutionary relationship distance. Besides ribosomal protein S3 (*rps3*), most introns are lost too quickly and lack the stability of protein-coding genes. The short RNA-seq reads from next-generation sequencing can improve the mitochondrial annotation accuracy and help study polycistronic transcripts and RNA processing. The transcription initiation sites may be located in the control region. Most introns do not serve as taxonomic markers and also lack open reading frames (ORFs). We assumed that the poly A tail was added to the polycistronic transcript before splicing and one polycistronic transcript (*trnM*_(1)_-*trnL*_(1)_-*trnA*-*trnF*-*trnK*-*trnL*_(2)_-*trnQ*-*trnH*-*trnM*_(2)_-*nad2*-*nad3*-*atp9*-*cox2*-*trnR*_(1)_-*nad4L*-*nad5*-*cob*-*trnC*-*cox1*-*trnR*_(2)_-*nad1*-*nad4*-*atp8*-*atp6*-*rns*-*trnY*-*trnD*-*trnS*-*trnN*-*cox3*-*trnG*-*nad6*-*trnV*-*trnI*-*trnS*-*trnW*-*trnP*-*rnl*(*rps3*)-*trnT*-*trnE*-*trnM*_(3)_) was first processed from the mitogenome and was subsequently processed into smaller mono-, di-, or tricistronic RNAs.

## Introduction

The genus *Beauveria* belonging to the family Cordycipitaceae, subclass Hypocreomycetidae, has a wide host range of insect species (Ghikas, Kouvelis & Typas, 2010), and is a cosmopolitan entomopathogenic fungal genus. (Ghikas, Kouvelis & Typas, 2010; Oda et al., 2014; Oh, Kong & Sung, 2015; Valero-Jiménez et al., 2016; Zhang et al., 2021). Approximately 30 strains of *Beauveria* were collected and sorted in the NCBI Taxonomy database (Schoch et al., 2020), and many unclassified *Beauveria* species were identified. Although easily distinguishable as a genus, most mitosporic fungal morphological characteristics are inadequate for species delimitation within a genus (Valero-Jiménez et al., 2016; Zhang et al., 2021). At present, an increasing number of *Beauveria* species genomes have been sequenced. However, the mitogenome of *Beauveria* species evolves faster than their nuclear genome (Valero-Jiménez et al., 2016). It contains introns and mobile elements, exhibits extensive polymorphisms, and has been increasingly used to examine genetic diversity within fungal populations (Valero-Jiménez et al., 2016). The first mitogenome of *the Beauveria* species (*B. bassiana*) (GenBank accession number: EU371503.2/NC_010652.2) was sequenced by Sanger sequencing and published in 2009 in the NCBI nucleotide database (Xu et al., 2009). It contains 2 rRNA, 25 tRNAs, and 15 protein-coding genes (PCGs) (Xu et al., 2009).

The mitogenome of *B*. *brongniartii* isolate IMBST95031 has an additional intron in *rnl*, differentiated from *B. bassiana* (Ghikas, Kouvelis & Typas, 2010). The mitogenomes of *B. pseudobassiana* strain C1010 (Oh, Kong & Sung, 2015), *B. malawiensis* isolate k89 (Glare et al., 2020), *B. caledonica* isolate fhr1 (Glare et al., 2020), and *B. lii* strain RCEF5500 (Zhang et al., 2021), especially *B. bassiana* (most abundant species of the genus *Beauveria*) provided insights into the mitogenome of the *Beauveria* genus (Ghikas, Kouvelis & Typas, 2010; Glare et al., 2020; Lee et al., 2017; Wang et al., 2020). Complete mitogenomes have high variations, and their lengths vary greatly (Uribe & Khachatourians, 2004). The minimum complete mitogenome length was 28,816 bp (*B. bassiana* isolate k4), whereas the maximum length was nearly double the minimum length, *i.e.,* 59,014 bp (*B. lii* strain RCEF5500). Complete mitogenomes of *Beauveria* species are haploid, maternally inherited, and have fewer introns, which can be easily sequenced and assembled using various sequencing techniques (Sanger sequencing, next-generation sequencing (NGS), and PacBio sequencing) (Wang et al., 2020; Xu et al., 2009; Zhang et al., 2021). However, since mitogenome annotations are mainly derived from similar species comparison and software prediction, and are not supported by RNA-seq transcripts data, it leads to problems with the accuracy of mitochondrial annotations and the inability to understand RNA processing. A complete and detailed transcription map of the *Dictyostelium discoideum* mitochondrial DNA revealed eight major polycistronic transcripts encoding polypeptides, ribosomal RNAs, interspersed transfer RNAs, and polycistronic transcripts processed into smaller transcripts in northern hybridization studies (Barth et al., 2001). PacBio sequencing has identified polycistronic transcripts, which are conserved and prevalent in several mushroom-forming fungal species, including *Plicaturopsis crispa* and *Phanerochaete chrysosporium* (Gordon et al., 2015). The PacBio full-length transcriptome profiling of insect mitochondrial gene expression investigates mitochondrial gene transcription, RNA processing, mRNA maturation, and several other related topics (Gao et al., 2016). However, a large number of transcriptomes sequenced by next-generation sequencing (NGS) are in the NCBI Sequence Read Archive (SRA) database, and their numbers are still increasing. The *Beauveria* strains transcriptome data shared by the NCBI SRA database has not adopted the PacBio or Oxford Nanopore techniques.

We sequenced both the genome and transcriptome of seven *Beauveria* strains using the Illumina sequencing platform in this study, and downloaded the SRA data (genome and transcriptome) of one *Beauveria* strain from the NCBI SRA database, which was sequenced using the Illumina sequencing platform in previous study (Valero-Jiménez et al., 2016). Although the NGS short reads resulted in incompletely assembled transcripts lacking some vital information (such as 5′ or 3′ end information) (Bai et al., 2021; Gao et al., 2016; Gordon et al., 2015), we built an analysis flow using NGS short reads to analyze mitogenome characteristics, polycistronic transcripts and RNA processing.

## Materials & Methods

### Fungal species

*B. amorpha* strain GYU-BMZ01 (BMZ072669), *B. amorpha* strain GYU-BMZ02 (BMZ072690), *B. brongniartii* strain GYU-BMZ03 (BMZ101344), *B. bassiana* strain GYU-BMZ04 (BMZ104186), *B. bassiana* strain YMM (CGMCC No. 11464), *B. caledonica* strain ATCC 64970, and *B. pseudobassiana* strain ATCC 90518 were selected for mitogenome sequencing. The *B. amorpha* strain GYU-BMZ01, *B. amorpha* strain GYU-BMZ02, *B. brongniartii* strain GYU-BMZ03, and *B. bassiana* strain GYU-BMZ04 (BMZ104186) were obtained from Ningbo Mingzhou Biotechnology Co., Ltd (Ningbo City, Zhejiang Province, China). *B. bassiana* strain YMM was isolated from *Alissonotum impressicolle* Arrow obtained from the Guangxi Key Laboratory of Biology for Crop Diseases and Insect Pests, Institute of Plant Protection, Guangxi Academy of Agricultural Sciences. *The B. bassiana* strain YMM was isolated from the odonatan larva of Guizhou University. *B. caledonica* strain ATCC 64970 and *B. pseudobassiana* strain ATCC 90518 were obtained from the American Type Culture Collection (ATCC). *B. bassiana* was cultivated on potato dextrose agar (PDA) for genomic DNA (gDNA) extraction. The surface of the PDA medium was scraped with a scalpel after 20 days of cultivation. We downloaded the SRA data (genome (SRR3269141) and transcriptome (SRR3269778–SRR3269780)) of *B. bassiana* strain ARSEF 8028 from the NCBI SRA database, which was sequenced using the Illumina sequencing platform in previous study (Valero-Jiménez et al., 2016).

### DNA extraction, library preparation and Illumina sequencing

Samples were collected from seven *Beauveria* strains and were subjected to genomic DNA extraction and quality check. The total DNA was extracted using a DNeasy Plant Maxi Kit (QIAGEN Inc., Valencia, CA, USA) following the kit handbook. The purity and concentration of the obtained gDNA were tested using a NanoPhotometer® spectrophotometer (Implen, CA, USA) and a Qubit® 2.0 fluorometer (Life Technologies, CA, USA), respectively. Sequencing libraries for the quality-checked gDNA were generated using a TrueLib DNA Library Rapid Prep Kit for Illumina sequencing (Illumina, Inc., CA, USA). The libraries were subjected to size distribution analysis using an Agilent 2100 bioanalyzer (Agilent Technologies, Inc., CA, USA), followed by real-time PCR quantitative test. The successfully generated libraries were sequenced using an Illumina NovaSeq 6000 platform (Illumina, Inc., San Diego, CA, USA), and 150-bp paired-end reads with an insert of approximately 350 bp were generated.

### RNA extraction, library preparation, and Illumina sequencing

Total RNAs from seven *Beauveria* were extracted using Trizol reagent (Life Technologies, Carlsbad, CA, USA) following the reagent handbook. The obtained RNA samples were subjected to RNase-free DNase I treatment to remove residual DNA. The purity, concentration, and integrity of the RNA were tested using a NanoDrop 2000 spectrophotometer (Implen, CA, USA), Aglient 2100 bioanalyzer (Agilent Technologies, Inc., Santa Clara, CA, USA), and Qubit 2.0 fluorometer (Life Technologies, Carlsbad, CA, USA), respectively. The mRNAs were purified from the total RNAs using poly-T oligo-attached magnetic beads and fragmented. Subsequently, cDNA libraries were generated for the quality-checked samples using a Tru Seq RNA Sample Prep Kit following the kit handbook. The successfully generated cDNA libraries were sequenced using using an Illumina NovaSeq 6000 platform (Illumina, Inc., CA, USA).

### DNA data cleaning and RNA data cleaning

The obtained raw reads were checked for sequence quality using fastqc version 0.11.9 (https://www.bioinformatics.babraham.ac.uk/projects/fastqc/) (Andrews, 2010), and filtered to obtain clean reads using fastp version 0.23.2 (https://github.com/OpenGene/fastp) (Chen et al., 2018). The quality control (QC) standards of reads from DNA were: (1) Trimming adapter sequences with >6 bases, (2) removing reads with >0 unidentified nucleotides (N), (3) Removing reads with >50% bases with Phred quality < Q20, (4) trimming 10 bases in front of the reads because these bases have a higher error rate, (5) trimming five bases in the tail region for reads resulting from bases with a higher error rate, (6) removing reads with <135 bases.

The QC standards for RNA reads were as follows: (1) Removing reads with >50% bases with Phred quality < Q10, (2) Removing reads with an average quality score of < 20, (3) trimming reads longer than 150 bases at their tail region to modify them to ≤ 150 bases, (4) trimming the poly-G tails of the reads, (5) filtering low-complexity reads (complexity is defined as the percentage of the base that differs from its next base); the threshold for the low-complexity filter is 10, which means that 10% complexity is required, and (6) Removing reads with < 80 bases.

### Mitogenome assembly and *de novo* transcriptome assembly

The mitogenomes of *the Beauveria* strains were assembled using NOVOPlasty version 4.3.1 (https://github.com/ndierckx/NOVOPlasty) (Dierckxsens, Mardulyn & Smits, 2016). The main software parameters were set as follows: (1) Mitogenome sequence of *B. bassiana* (GenBank accession number: EU371503.2/NC_010652.2) (Xu et al., 2009) was selected as the seed sequence and was indirectly extended using NOVOPlasty. (2) Because the length of the mitogenome sequence of *Beauveria* varies greatly, the expected mitogenome size was set to range from 20,000 to 40,000 bp. (3) The value of K-mer (33) was the default parameter.

**Figure 1:**
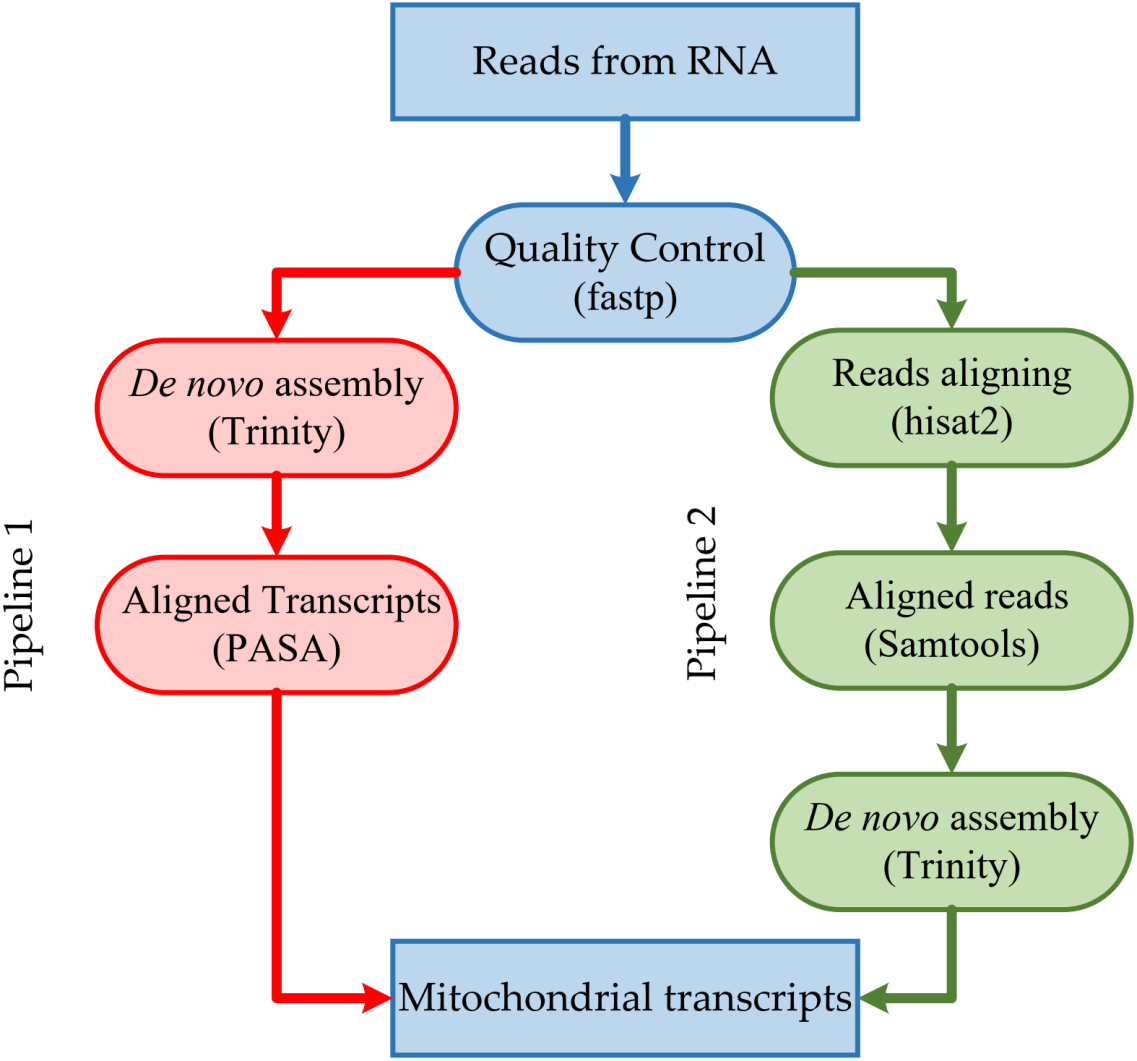
Overview of the steps in the pipeline for mitochondrial transcripts.

The resulting high-quality cleaned reads from RNA-seq were assembled *de novo* into transcripts using Trinity version 2.13.2 (https://github.com/trinityrnaseq/trinityrnaseq/) (Grabherr et al., 2011) with default parameters (Fig. 1). The resulting high-quality cleaned reads from RNA-seq were aligned to the corresponding mitogenome sequences using HISAT2 version 2.2.1 (https://github.com/DaehwanKimLab/hisat2) (Kim et al., 2019) with default parameters (Fig. 1). Aligned short reads were extracted using SAMtools version 1.14 (https://github.com/samtools/samtools) (Danecek et al., 2021) and assembled *de novo* into transcripts using Trinity version 2.13.2 (Grabherr et al., 2011), with default parameters.

### Mitogenome annotation and characteristics analysis

Using Perna and Kocher’s formula (Perna & Kocher, 1995), the AT-skew ((A − T)/(A + T)) and GC-skew ((G − C)/(G + C)) of the nucleotide sequences of mitogenomes were estimated to investigate the nucleotide composition bias. Using the mitogenome sequences of *Cordyceps bassiana* isolate Bb147 (EU100742.1) (Ghikas, Kouvelis & Typas, 2010), *B. pseudobassiana* strain C1010 (KF297618.1/NC_022708.1) (Oh, Kong & Sung, 2015), *B. caledonica* isolate fhr1 (KT201150.1/NC_030636.1) (Glare et al., 2020), *B. bassiana* isolate k4 (KT201148.1) (Glare et al., 2020), *Cordyceps brongniartii* isolate IMBST95031 (EU100743.1/NC_011194.1) (Ghikas, Kouvelis & Typas, 2010), and *B. bassiana* (NC_010652.2) (Xu et al., 2009) as reference sequences, the mitogenomes were initially annotated using GeSeq Version 2.03 (https://chlorobox.mpimp-golm.mpg.de/geseq.html) (Tillich et al., 2017), including the third-party software tRNAscan-SE version 2.0.7 (Chan & Lowe, 2019), ARAGORN version 1.2.38 (Laslett & Canback, 2004), and BLAT v36 × 7 (Kent, 2002). To improve mitogenome annotation and correct prediction errors, we annotated mitogenomes using the trinity-assembly transcripts using PASA version 2.5.1 (https://github.com/PASApipeline/PASApipeline) (Haas et al., 2003), including third-party software GMAP version 2021.08.25 (http://research-pub.gene.com/gmap/) (Wu & Watanabe, 2005), PBLAT version 2.5 (https://github.com/icebert/pblat) (Wang & Kong, 2019) (including BLAT version 36 (Kent, 2002)), and minimap2 version 2.24 (https://github.com/lh3/minimap2) (Li, 2018). Comparative mitogenome analysis was performed using Mauve 2.4.0 software (Darling et al., 2004) and mVISTA viewer (Dubchak et al., 2000; Frazer et al., 2004) with LAGAN (Brudno et al., 2003). The sequences of the transcripts were aligned to mitogenome sequences using BLASTN of BLAST+ 2.13.0 (Zhang et al., 2000) with MegaBLAST (Morgulis et al., 2008) on the NCBI web.

### Phylogenetic analysis

Phylogenetic relationships of the genus *Beauveria* were deduced using MEGA 11 software (Tamura, Stecher & Kumar, 2021) with 15 amino acid sequences of 16 species using the maximum likelihood (ML) method. Fourteen mitogenomes were obtained from *Beauveria* strains. The mitogenomes of *Metarhizium anisopliae* strain ME1 and *Trichoderma reesei* were selected as the outgroups. The amino acid sequences of 15 PCGs of each mitogenome were connected end to end in the order of *rps3*-*nad2*-*nad3*-*atp9*-*cox2*-*nad4L*-*nad5*-*cob*-*cox1*-*nad1*-*nad4*-*atp8*-*atp6*-*cox3*-*nad6* to form a sequence. For ML, models with the lowest Bayesian information criterion (BIC) scores were considered to best describe the substitution pattern. Based on BIC = 50395.840, the general reversible chloroplast (cpREV) (Adachi et al., 2000) + gamma distribution (G) + amino acid frequencies (F) model was chosen as the optimal evolutionary model with 1000 bootstrap replications for the phylogenetic analysis. Initial trees for the heuristic search were obtained automatically by applying the neighbor-join and BioNJ algorithms to a matrix of estimated pairwise distances and then selecting the topology with a superior log-likelihood value. The percentage of replicate trees in which the associated taxa clustered together in the bootstrap test of 1000 replicates is shown above the branches (Felsenstein, 1985). There were a total of 5572 positions in the final dataset.

### RNA reverse transcription, PCR amplification and sanger sequencing

RNA was reversed to cDNA using the Kit PrimeScript™ RT Reagent Kit with gDNA Eraser (Perfect Real Time) (No. RR047A; TaKaRa). According to the target region, primers were designed using Primer3 version 2.6.1 (Koressaar & Remm, 2007), and PCR amplification was performed using GeneExplorer TC-XP-D (Hangzhou Bioer Technology Co., Inc., Hangzhou, CN). 2 µl of PCR products were used for electrophoresis on 1% agarose gel. PCR products were sequenced using an ABI 3730 automatic sequencer (Applied Biosystems, Foster City, CA, USA).

## Results

### Mitogenome and RNA assembly

**Figure 2:**
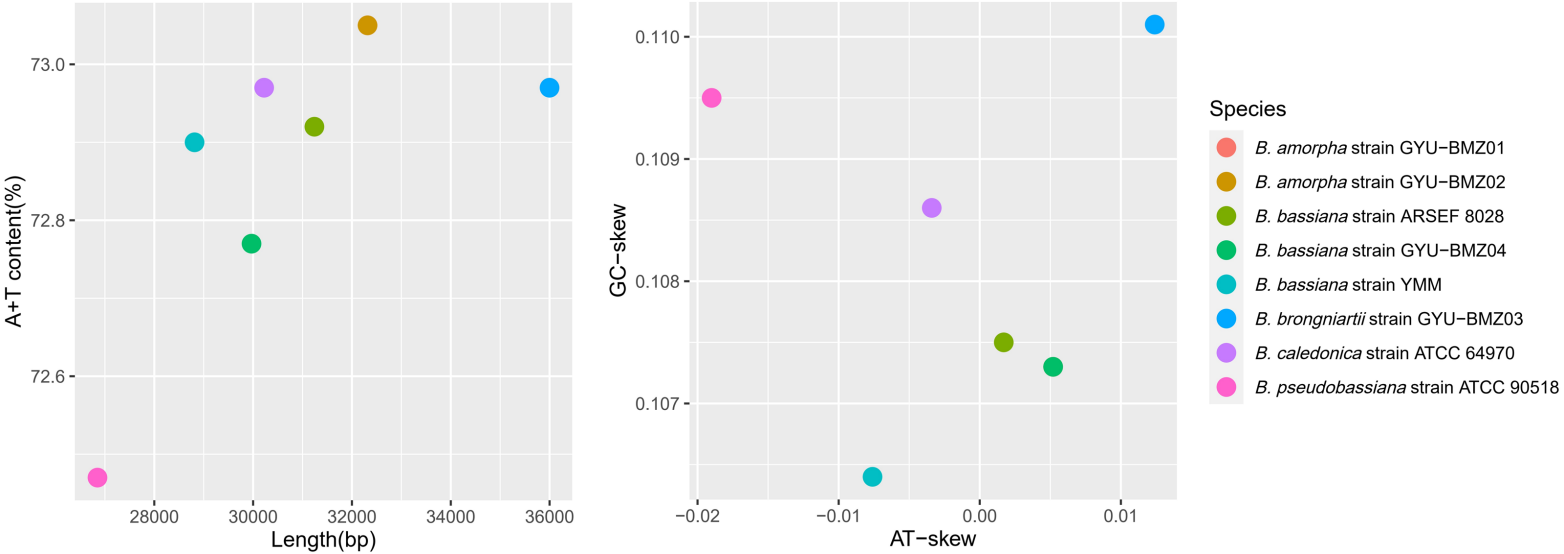
Scatter-plot charts representing various characteristics of the mitogenomes from 8 *Beauveria* species. The scatter-plot charts of the (A) length and A + T content, and (B) AT- and GC-skew of the mitogenomes from the 8 *Beauveria* species. The different colors of the dots represent the different species, as indicated in the figure key.

These high-quality short reads define eight complete mitogenomes with 100% coverage at high average read depths (from 588 times for *B. caledonica* strain ATCC 64970 to 3151 times for *B. bassiana* strain ARSEF 8028), comprising a typical single circular DNA molecule with a total length ranging from 26,850 (*B. caledonica* strain ATCC 64970) to 35,999 bp (*B. brongniartii* strain GYU-BMZ03) (Fig. 2A and Table S1). *Beauveria* demonstrated an obvious AT bias, with AT content ranging from 72.47% (*B. caledonica* strain ATCC 64970) to 73.05% (*B. amorpha* strain GYU-BMZ01 and GYU-BMZ02) (Fig. 1A and Table S1), and the AT-skew ranging from −0.0190 (*B. caledonica* strain ATCC 64970) to 0.0124 (*B. brongniartii* strain GYU-BMZ03) (Fig. 2B and Table S1). However, the GC-skew was consistently positive (from 0.1064 for *B. bassiana* strain YMM to 0.1101 for *B. brongniartii* strain GYU-BMZ03) (Fig. 2B and Table S1).

**Figure 3:**
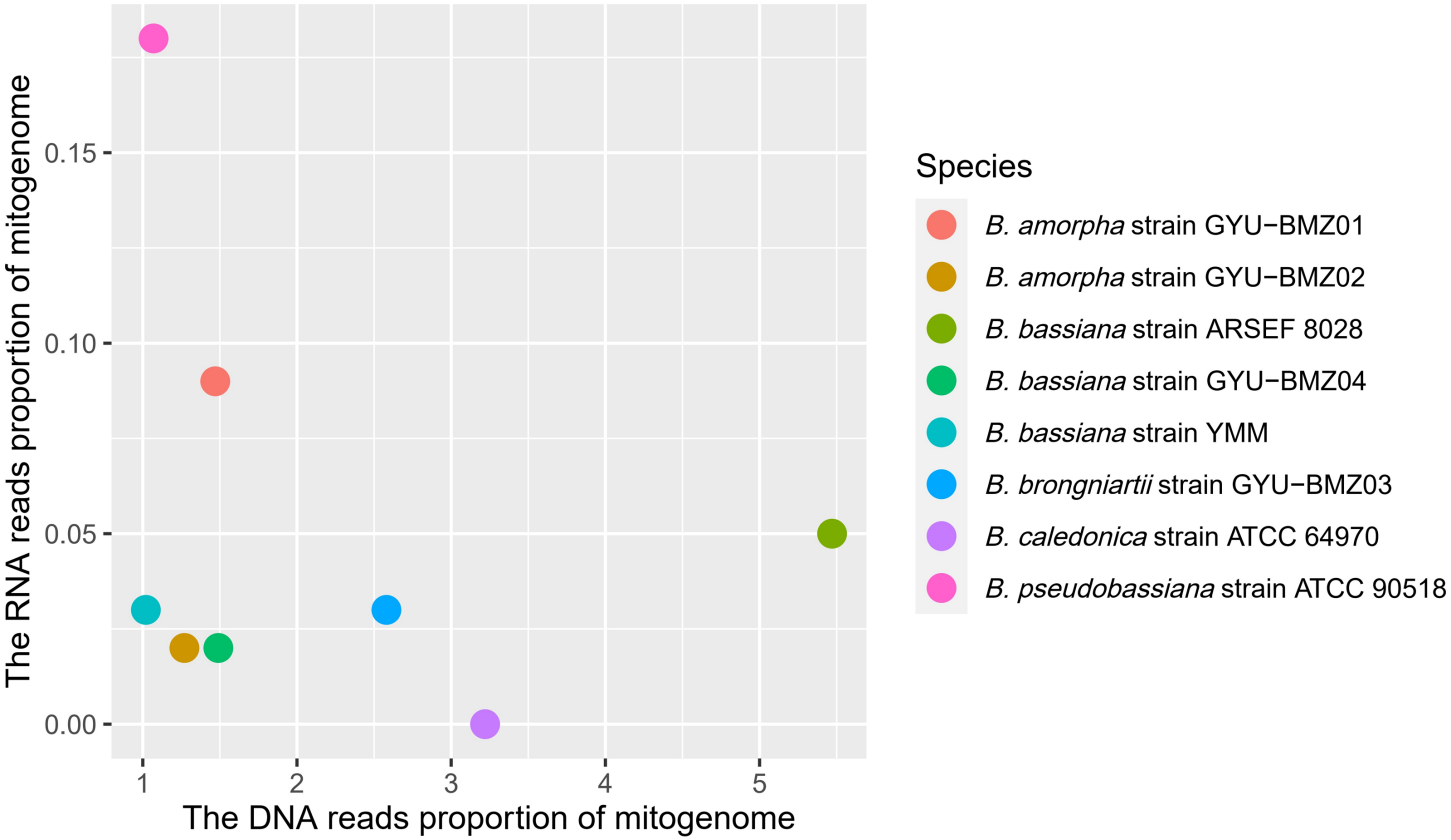
Scatter-plot chart of the DNA reads proportion and RNA reads proportion of mitogenomes from 8 *Beauveria* species. The different colors of dots represent the different species.

Although the proportion of short reads of the mitogenomes in the total reads of DNA ranged from 1.07% (*B. caledonica* strain ATCC 64970) to 5.47% (*B. bassiana* strain ARSEF 8028), those from the RNA accounted for <0.18% of the total short reads, especially the mitochondrial RNA read proportion of *B. caledonica* strain ATCC 64970 being almost zero (Fig. 3). This may have something to do with being in a closed petri dish.

### Mitogenome annotation

The conserved genes of the *Beauveria* species mitogenome were located on the positive strand and in the same order and orientation as *B*. *bassiana* (NC_010652.2) (Xu et al., 2009), which encodes fifteen protein-coding genes (PCGs) that include the ribosomal protein S3 (*rps3*), seven NADH dehydrogenase subunits (*nad1*-*6* and *nad4L* genes), three cytochrome oxidase subunits (*cox1*-*3*), apocytochrome b (*cob*), three subunits of ATP synthase (*atp6*, *8*, *9*), small and large subunit ribosomal RNA genes (*rnl* and *rns*), 25 tRNAs that include three tRNAs for methionine, two for arginine, two for leucine, two for serine, and only one tRNA for other amino acids, and one control region located between *trnM*_(3)_ and *trnM*_(1)_. Transcriptome annotation revealed that *rnl* was longer than the reference mitogenome (NC_010652.2), extending 30 bp forward and 10 bp backward. These regions have multiple AT repeats, possibly circularizing the *rnl* gene. We also observed some reads of the *B. amorpha* strain GYU-BMZ01 and *B*. *pseudobassiana* strain ATCC 90518 from RNA-seq with the half bases aligned at the head of *rnl* and half bases aligned at its tail, thus causing errors in the *de novo* assembly of the transcriptome. We found a read in *the B. amorpha* strain GYU-BMZ02 showing that the *rnl* gene has a polyA tail at the 3′ end.

**Figure 4:**
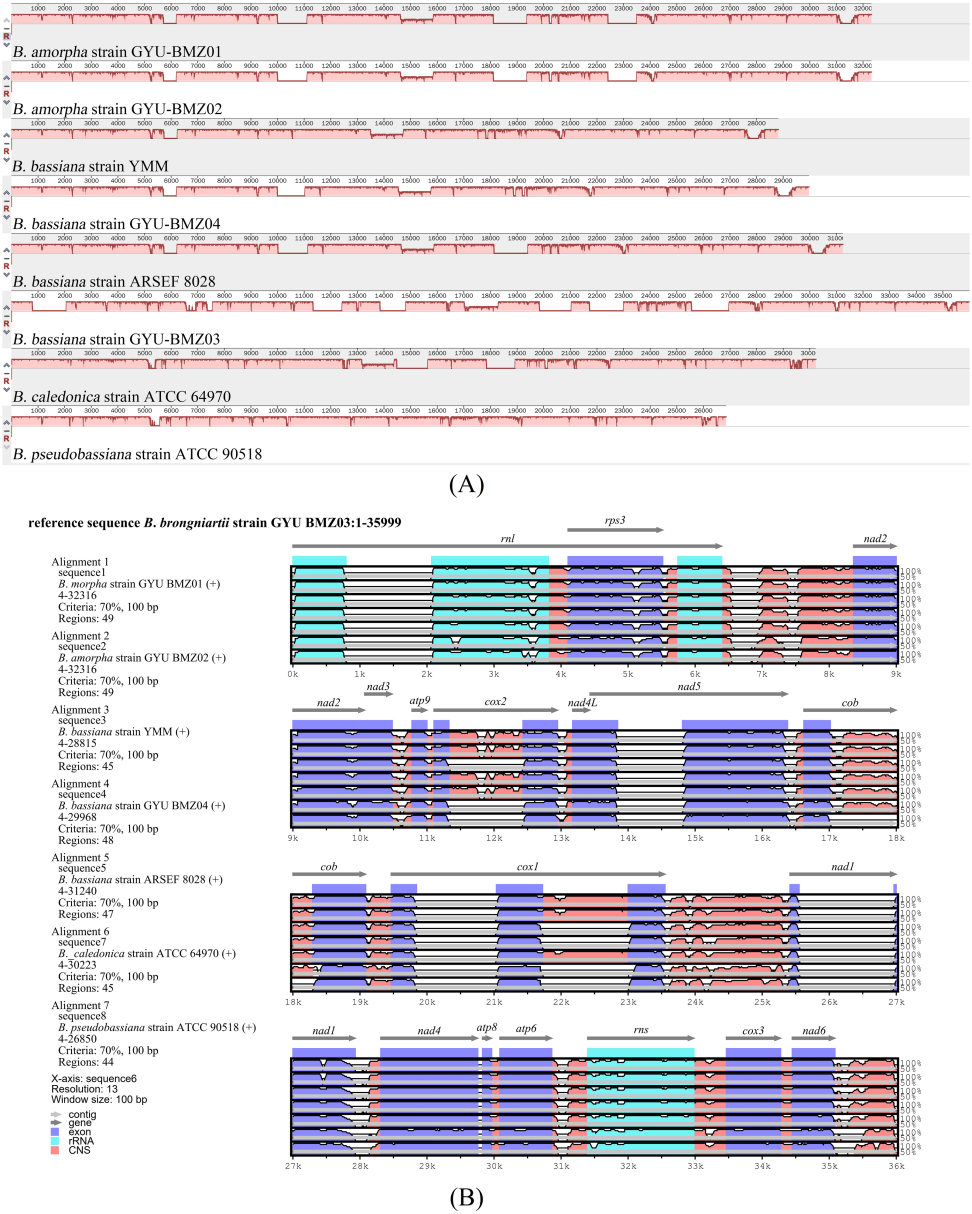
Visualization of the alignment of mitogenome sequences. (A) Mauve-based identity plots. Numbered scale bars indicate the distance in base pairs, and the vertical bars indicate the sequence similarity. Size differences among eight mitogenomes were mainly due to the presence/absence of introns in certain genes. (B) mVISTA-based identity plots showing sequence identity between the eight sequenced *Beauveria* species mitogenomes with the *B. brongniartii* strain GYU-BMZ03 as a reference. Genome regions are color-coded as contig, gene, rRNA coding, exon, or conserved noncoding sequences (CNS).

**Figure 5:**
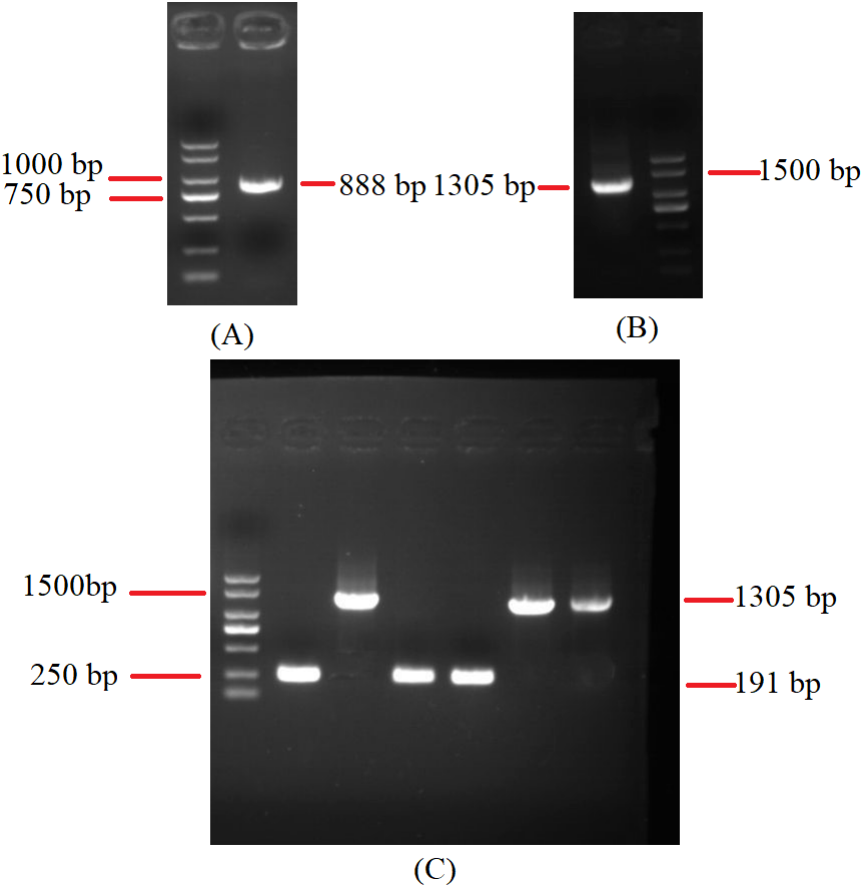
Agarose gel electrophoresis of PCR products of *cox2*. (A) Agarose gel electropherogram of cDNA PCR product of *cox2* in mitogenome of *B. bassiana* strain GYU-BMZ04. (B) Agarose gel electropherogram of DNA PCR product of *cox2* in mitogenome of *B. bassiana* strain GYU-BMZ04. (C) Agarose gel electropherogram of DNA PCR product of *cox2* for six *Beauveria* strains, which were *B. bassiana* strain YMM, *B. amorpha* strain GYU-BMZ01 , *B. caledonica* strain ATCC 64970, *B. pseudobassiana* strain ATCC 90518 , *B. bassiana* strain GYU-BMZ04, and *B. brongniartii* strain GYU-BMZ03 from left to right. The markers were from the DL2000 Plus DNA Marker, which from top to bottom are 2,000, 1,500, 1,000, 750, 500, 250, 100 bp.

To investigate the extent of genome divergence, we performed multiple alignments of the eight *Beauveria* mitogenome sequences (Fig. 4). The results revealed high sequence similarity across all the eight *Beauveria* mitogenomes for coding DNA sequence (CDS), with some structural variations existing in the mitogenomes. We observed mitogenome length differences mostly in the introns (Fig. 4 and Table 1). The number of introns ranged from one (*B. pseudobassiana* strain ATCC 90518) to eight (*B. brongniartii* strain GYU-BMZ03), and were located in *rnl*, *cob*, *cox1*, *cox2*, and *nad1* (Fig. 4 and Table 1). The total length of the introns varied from 1,882 bp (*B. pseudobassiana* strain ATCC 90518) to 10,422 bp (*B. brongniartii* strain GYU-BMZ03), with their contribution to the respective total mitogenome size ranging from 7.01% (*B. pseudobassiana* strain ATCC 90518) to 28.95% (*B. brongniartii* strain GYU-BMZ03). The intron of *rnl* (the second intron of *rnl* for *B. brongniartii* strain GYU-BMZ03) was conserved and contained an open reading frame (ORF) for *rps3*. Introns in other genes are thought to contain ORFs (Ghikas, Kouvelis & Typas, 2010; Oh, Kong & Sung, 2015; Zhang et al., 2021), including the LAGLI-DADG and GIY-YIG endonuclease located in the *cox1* and *nad1* introns (Ghikas, Kouvelis & Typas, 2010), respectively. After comparing the eight *Beauveria* strains, we concluded that there were no ORFs owing to the loss of introns among the different species. Even if these intron sequences were not lost, there were many SNP mutations, and even the start codon could not be identified (*B. bassiana* strain YMM and *B. bassiana* strain GYU-BMZ04). The *cox2* gene of *B. brongniartii* has one intron, including a putative GIY-YIG homing endonuclease (Oh, Kong & Sung, 2015). However, the *cox2* gene of *B. pseudobassiana* strain ATCC 90518 did not contain any introns from the mitogenome and transcript sequences. The intron types of *Beauveria* species mitogenomes were group I introns (Wang et al., 2020; Zhang & Zhang, 2019), of which the boundaries were marked by a U residue at the 3′ end of upstreaming exon and a G residue at the 3′ end of the intron (Burke, 1988; Cech, 1988; Saldanha et al., 1993). The splicing model was mainly GT-AG in introns mainly of *Beauveria* species mitogenomes (Wang et al., 2020). For verification, the introns of the *cox2* can be shown using cDNA and PCR in mitogenome of *B. bassiana* strain GYU-BMZ04 (Fig. 5, Files S1 and S2). The *cox2* gene contained one intron in in mitogenome of *B. bassiana* strain GYU-BMZ04, but the *cox2* transcript did not contain intron. The *cox2* gene of *B. amorpha* strain GYU-BMZ01, *B. bassiana* strain GYU-BMZ04, and *B. brongniartii* strain GYU-BMZ03 contained intron, which is consistent with the results of assembly and annotation.

The mitogenomes contained 39 intergenic and two overlapping regions. The total length of the intergenic regions ranged from 5198 bp (*B. pseudobassiana* strain ATCC 90518) to 6092 bp (*B. bassiana* strain GYU-BMZ04), thus contributing to a total mitogenome size ranging from 15.76% (*B. brongniartii* strain GYU-BMZ03) to 20.33% (*B. bassiana* strain GYU-BMZ04). The A + T content was similar for these regions in the mitogenomes (∼74.5%), with the largest intergenic region being between *cox1* and *trnR*-UCU. Overlapping regions of 1 bp were located between both *nad2* and *nad3*, and between *nad4L* and *nad5*.

### Phylogenetic analysis

**Figure 6:**
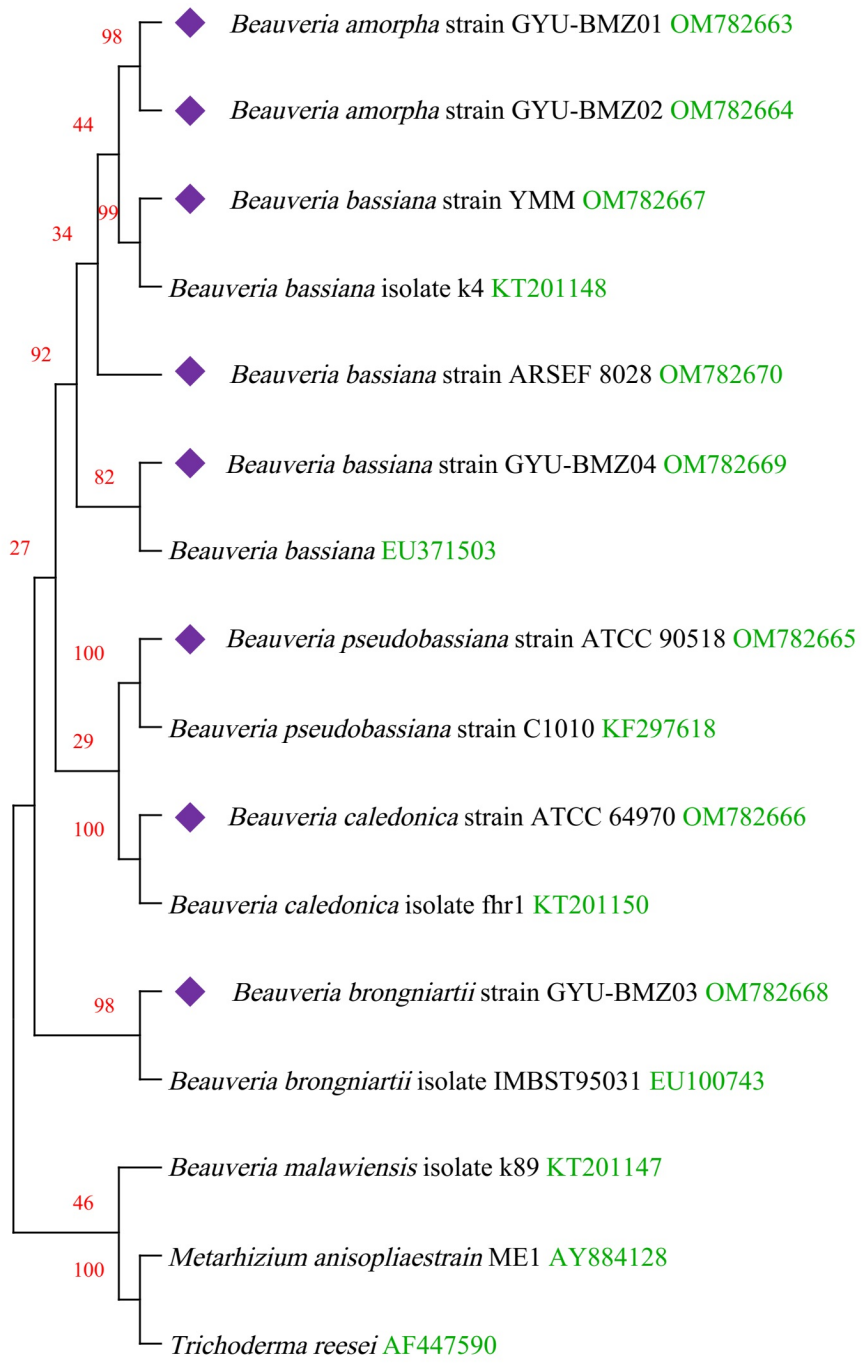
Maximum-likelihood phylogenetic tree based on the 15 protein sequences of mitogenomes from 16 species. Branches corresponding to partitions reproduced in less than 50% bootstrap replicates are collapsed. The percentage of replicate trees, in which the associated taxa are clustered together in the bootstrap test of 1,000 replicates, are shown above the branches in red. The complete mitogenome of *Beauveria* from this study is indicated in purple. GenBank accession numbers are indicated in green.

We obtained fourteen mitogenomes from the *Beauveria* species and selected the mitogenome of *Metarhizium anisopliae* strain ME1 and *Trichoderma reesei* as outgroups. Based on the lowest BIC value (50395.840), we constructed a phylogenetic tree using the maximum likelihood (ML) method and a general reversible chloroplast (Adachi et al., 2000) + amino acid frequencies (F) + gamma distribution (G) model with 1000 bootstrap replications (Fig. 6). The structure of the phylogenetic tree is similar to reported in previous studies (Glare et al., 2020; Zhang et al., 2021). The phylogenetic position of *B. amorpha* within the *Beauveria* was first evaluated using complete mitogenomes, and the results showed that *B. amorpha* was closed to *B. bassiana*. *B*. *pseudobassiana* and *B. caledonica* were on the same branch. The presence or absence of mitochondrial introns cannot serve as a taxonomic marker (Glare et al., 2020), which also verifies that most introns lack the stability of coding regions from another aspect. All genes in the *Beauveria* species are on the same chain, which might result in changes to the intron number.

**Table 1:** The number of introns in mitochondrial genes.

**Species**	***rnl***	***cox2***	***nad5***	***cob***	***cox1***	***nad1***	**Total**
*B. amorpha* strain GYU-BMZ01	1	1	0	1	1	1	5
*B. amorpha* strain GYU-BMZ02	1	1	0	1	1	1	5
*B. bassiana* strain YMM	1	0	0	1	0	0	2
*B. bassiana* strain GYU-BMZ04	1	1	0	1	0	0	3
*B. bassiana* strain ARSEF 8028	1	1	0	1	1	0	4
*B. brongniartii* strain GYU-BMZ03	2	1	1	1	2	1	8
*B. caledonica* strain ATCC 64970	1	0	0	2	1	0	4
*B. pseudobassiana* strain ATCC 90518	1	0	0	0	0	0	1

## Discussion

We adopted two annotation processes (Fig. 1): (1) we aligned the reads to the mitogenome, extracted the aligned reads, conducted *de novo* assembly, and then mapped transcripts to the mitogenome, and (2) we performed the *de novo* assemble and mapping of the transcripts to the mitochondrial genome. There are differences between mitochondrial transcripts and nuclear transcripts. While nuclear transcripts consist of a very high proportion of the entire transcriptome, we reduced the computational resources used during *de novo* assembly by filtering reads of nuclear transcripts. Although the first process was much more efficient than the second, it filtered out reads during their alignment, *i.e.,* reads of *B. amorpha* strain GYU-BMZ01 and *B*. *pseudobassiana* strain ATCC 90518 from RNA-seq with the half bases aligned the head of *rnl,* and half bases aligned the tail. Although NGS short reads resulted in incompletely assembled transcripts that lack some vital information (*e.g.*, 5′ or 3′ end information) (Bai et al., 2021; Gao et al., 2016; Gordon et al., 2015) and do not accurately determine the continuity of the transcript, we can make predictions with different types of reads, which can improve the utilization of NGS data and aid in the mitogenome annotation.

### Polycistronic transcripts and RNA processing

**Figure 7:**
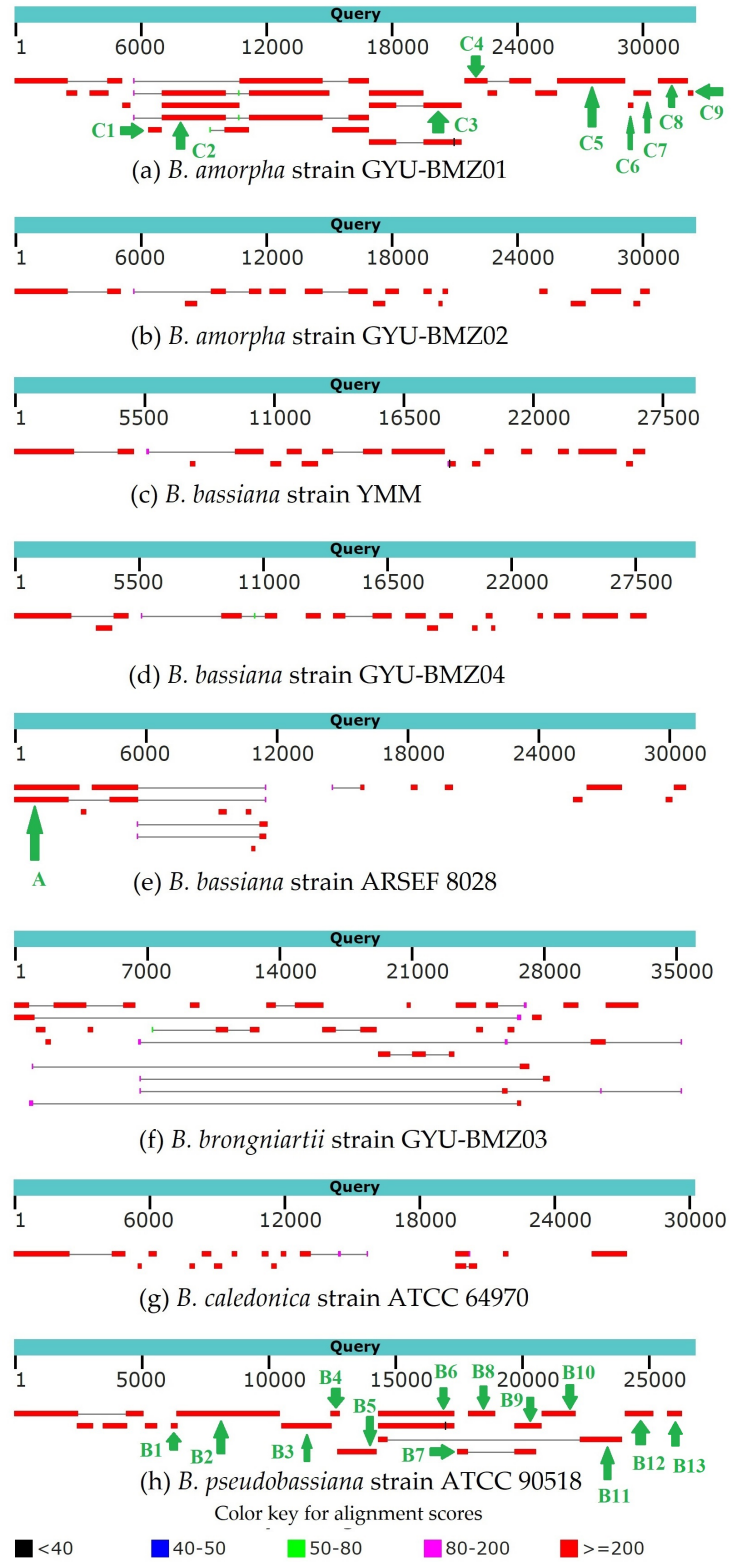
(A–H) BLAST graphic depicting transcript sequences aligned to the query sequence. Separate aligned regions on the same sequence are connected by a thin grey line.

**Figure 8:**
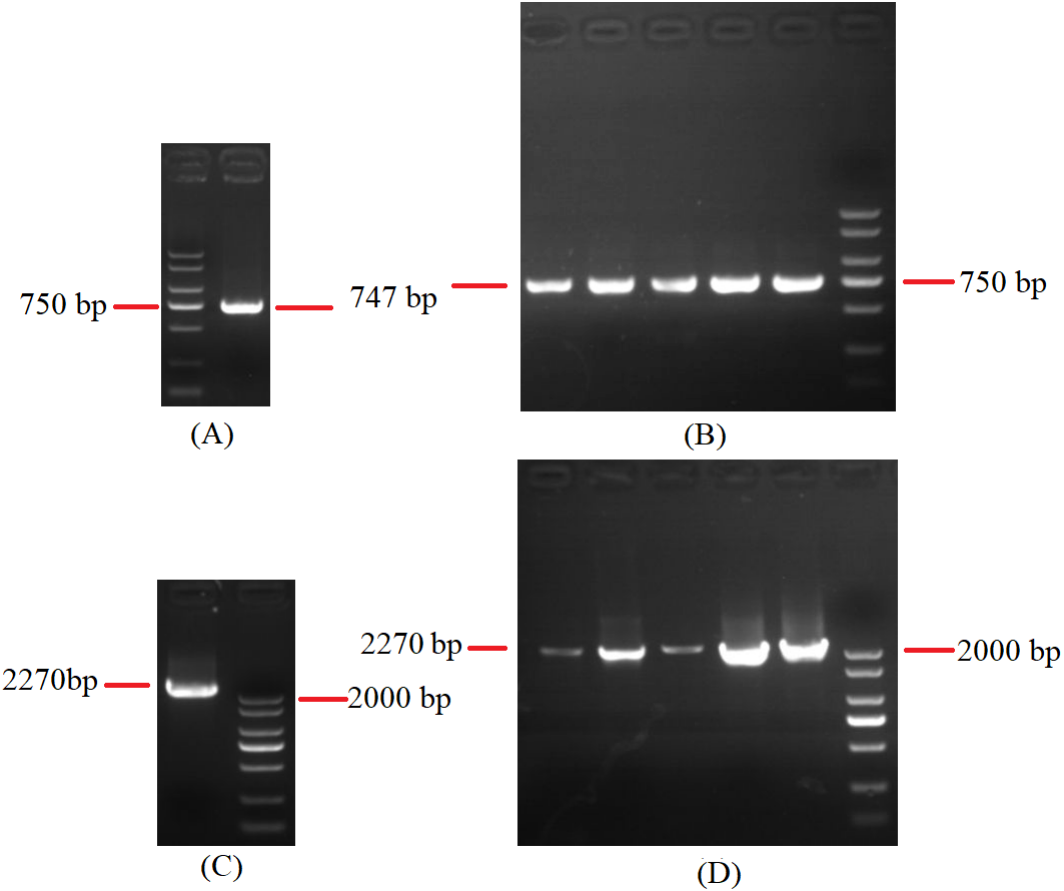
Agarose gel electropherogram of cDNA PCR product of *rns*-*trnY*-*trnD*-*trnS*-*trnN*-*cox3* and *cox1*-*trnR*_(2)_-*nad1*. (A) Agarose gel electropherogram of cDNA PCR product of *rns*-*trnY*-*trnD*-*trnS*-*trnN*-*cox3* in mitogenome of *B. bassiana* strain GYU-BMZ04. (B) Agarose gel electropherogram of cDNA PCR product of *rns*-*trnY*-*trnD*-*trnS*-*trnN*-*cox3* for five *Beauveria* strains, which were *B. amorpha* strain GYU-BMZ01, *B. caledonica* strain ATCC 64970, *B. brongniartii* strain GYU-BMZ03, *B. pseudobassiana* strain ATCC 90518, and *B. bassiana* strain YMM from left to right. (C) Agarose gel electropherogram of cDNA PCR product of *cox1*-*trnR*_(2)_-*nad1* in mitogenome of *B. bassiana* strain GYU-BMZ04. (D) Agarose gel electropherogram of cDNA PCR product of *cox1*-*trnR*_(2)_-*nad1* for five *Beauveria* strains, which were *B. amorpha* strain GYU-BMZ01, *B. caledonica* strain ATCC 64970, *B. brongniartii* strain GYU-BMZ03, *B. pseudobassiana* strain ATCC 90518, and *B. bassiana* strain YMM from left to right. The markers were from the DL2000 Plus DNA Marker, which from top to bottom are 2,000, 1,500, 1,000, 750, 500, 250, 100 bp.

Genome-wide polycistronic transcripts are prevalent and conserved among mushroom-forming Agaricomycetes (Gordon et al., 2015). Most polycistronic transcripts are subsequently processed into smaller mono-, di-, or tricistronic RNAs (Barth et al., 2001). In this study, we found two types of reads for regions between introns and exons. The first type of reads were splicing reads, which could be mapped to two adjacent exons, while the second type could be mapped to introns, exons, and across the boundary. Therefore, we can classify *de novo* assembled transcripts into (1) intron-free and (2) intron-containing transcripts. mRNA was purified from the total RNA using poly T oligo-attached magnetic beads. Mature mRNAs with poly A tails were intron-free. Although some introns may contain ORFs, the origin and termination sites of genes located within introns are hundreds of bases away from the exon region. There should not be any second type of reads. Owing to the “efficiency of splicing”, (Darnell, 2013), we propose that the maturation processing of pre-mRNA involves polyadenylation, splicing, and cleavage. The poly A tail was added to the polycistronic transcript before splicing. For the *B. amorpha* strain GYU-BMZ01, the RNA-seq read coverage was high (up to 94.72%), and with the addition of some tRNA regions that are not reads, the coverage will be even higher. Based on the transcript alignment results (Fig. 7 and Table 2), some polycistronic transcripts were likely present. (1) The first region is *rnl* (*rps3*)-*trnT*-*trnE*-*trnM*_(3)_. We found contig A that exists in *B. bassiana* strain ARSEF 8028 (Fig. 7E and Table 2); therefore, a polycistronic transcript of *rnl* (*rps3*)-*trnT*-*trnE*-*trnM*_(3)_ is present. (2) The second region is *trnM*_(1)_-*trnL*_(1)_-*trnA*-*trnF*-*trnK*-*trnL*_(2)_-*trnQ*-*trnH*-*trnM*_(2)_-*nad2*-*nad3*-*atp9*-*cox2*-*trnR*_(1)_-*nad4L*-*nad5*-*cob*-*trnC*-*cox1*-*trnR*_(2)_-*nad1*-*nad4*-*atp8*-*atp6*-*rns*-*trnY*-*trnD*-*trnS*-*trnN*-*cox3*-*trnG*. Owing to defects in the Trinity software assembly, some overlapping contigs such as contigs B3 and B4, were not assembled together (Fig. 7H and Table 2). Some contigs, like contig B4 and contig B5 (Fig. 7H and Table 2), were located at the beginning and end of the coding region of the same gene, despite not being connected. After combining B1-B12 and C1-C9, several continuous fragments like *trnL*_(1)_-*trnA*-*trnF*-*trnK*-*trnL*_(2)_*-trnQ-trnH-trnM*_(2)_*-nad2-nad3-atp9-cox2-trnR*_(1)_*-nad4L-nad5-cob-trnC-cox1*, *nad1-nad4-atp8-atp6-rns-trnY*, and *trnD-trnS-trnN-cox3* were formed. Some tRNAs did not contain reads, such as *trnM*_(1)_, *trnR*_(2)_, *trnG*, and *trnP*. The tRNAs preceding mRNAs or rRNAs in the polycistronic transcripts were removed by 30–50 cleavages (Gao et al., 2016), whereas some tRNAs were cleaved, or were not sequenced because of limitations in the sequencing technology. *trnM*_(1)_ is 2 bp apart from *trnL*_(1)_, therefore, it might be *trnM*_(1)_-*trnL*_(1)_-*trnA*-*trnF*-*trnK*-*trnL*_(2)_-*trnQ*-*trnH*-*trnM*_(2)_-*nad2*-*nad3*-*atp9*-*cox2*-*trnR*_(1)_-*nad4L*-*nad5*-*cob*-*trnC*-*cox1*. *trnG* began at base 25,199 on the mitogenome sequence of *B. pseudobassiana* strain ATCC 90518, with contig B12 ending at base 25,172, which is 26 bp apart from *trnG* (Fig. 7H and Table 2). Therefore, it may be *trnD*-*trnS*-*trnN*-*cox3*-*trnG*. Since *trnY* is 3 bp apart from *trnD*, *nad1*-*nad4*-*atp8*-*atp6*-*rns*-*trnY* and *trnD*-*trnS*-*trnN*-*cox3* were merged into *nad1*-*nad4*-*atp8*-*atp6*-*rns*-*trnY*-*trnD*-*trnS*-*trnN*-*cox3*-*trnG*. For verification, *rns*-*trnY*-*trnD*-*trnS*-*trnN*-*cox3* can be shown using cDNA and PCR in mitogenome of *B. bassiana* strain GYU-BMZ04 (Figs. 8A and 8B, Files S1 and S3). The coding region of *trnR*_(2)_ ranged from 17,359–17,429 in the mitogenome sequence of *B. pseudobassiana* strain ATCC 90518, located between *cox1* and *nad1*. Since the distance between contig B6 and contig B7 was exactly the length of one *trnR*_(2)_ (Fig. 7H and Table 2). For verification, *cox1*-*trnR*_(2)_-*nad1* can be shown using cDNA and PCR in mitogenome of *B. bassiana* strain GYU-BMZ04 (Figs. 8C and 8D, Files S1 and S4). We merged *trnM*_(1)_-*trnL*-*trnQ*-*trnH*-*trnM*_(2)_-*nad2*-*nad3*-*atp9*-*cox2*-*trnR*_(1)_-*nad4L*-*nad5*-*cob*-*trnC*-*cox1* and *nad1*-*nad4*-*atp8*-*atp6*-*rns*-*trnY*-*trnD*-*trnS*-*trnN*-*cox3*-*trnG* into a polycistronic transcript, *trnM*_(1)_-*trnL*_(1)_-*trnA*-*trnF*-*trnK*-*trnL*_(2)_-*trnQ*-*trnH*-*trnM*_(2)_-*nad2*-*nad3*-*atp9*-*cox2*-*trnR*_(1)_-*nad4L*-*nad5*-*cob*-*trnC*-*cox1*-*trnR*_(2)_-*nad1*-*nad4*-*atp8*-*atp6*-*rns*-*trnY*-*trnD*-*trnS*-*trnN*-*cox3*-*trnG*. (3) The third region is *nad6*-*trnV*-*trnI*-*trnS*-*trnW*-*trnP*. According to contigs B13, C8, and C9 (Figs. 7A, 7H and Table 2), *trnV* and *trnI* both only sequenced part of the sequence; contig C8 is 53 bp apart from C9, but *trnV* is 2 bp apart from *trnI*, so there is a polycistronic transcript of *nad6*-*trnV*-*trnI*-*trnS*-*trnW*-*trnP*.

**Table 2:** Position, length and gene content of polycistronic transcripts for the *B. bassiana* strain ARSEF 8028, *B. pseudobassiana* strain ATCC 90518 and *B. amorpha* strain GYU-BMZ01.

**Contig**	**Positon**	**Species**	**Gene content**
A	1–5,664	*B. bassiana* strain ARSEF 8028	*rnl* (*rps3*)-*trnT*-*trnE*-*trnM3*
B1	6,198–6,433	*B. pseudobassiana* strain ATCC 90518	*trnL1*-*trnA*-*trnF*-*trnK*-*trnL2*
B2	6,414–10,490	*B. pseudobassiana* strain ATCC 90518	*trnL2*-*trnQ*-*trnH*-*trnM2*-*nad2*-*nad3*-*atp9*-*cox2*-*trnR1*
B3	10,540–12,490	*B. pseudobassiana* strain ATCC 90518	*nad4L*-*nad5*
B4	12,470–12,830	*B. pseudobassiana* strain ATCC 90518	*nad5*-*cob*
B5	12,729–14,308	*B. pseudobassiana* strain ATCC 90518	*cob*-*trnC*
B6	14,313–17,358	*B. pseudobassiana* strain ATCC 90518	*cox1*
B7	17,429–17,866	*B. pseudobassiana* strain ATCC 90518	*nad1*
B8	17,862–18,968	*B. pseudobassiana* strain ATCC 90518	*nad1*
B9	19,715–20,804	*B. pseudobassiana* strain ATCC 90518	*nad1*-*nad4*-*atp8*
B10	20,795–22,128	*B. pseudobassiana* strain ATCC 90518	*atp8*-*atp6*
B11	22,273–23,966	*B. pseudobassiana* strain ATCC 90518	*rns*-*trnY*
B12	24,047–25,172	*B. pseudobassiana* strain ATCC 90518	*trnS*-*trnN*-*cox3*
B13	25,704–26,318	*B. pseudobassiana* strain ATCC 90518	*nad6*-*trnV*
C1	6,367–6,996	*B. amorpha* strain GYU-BMZ01	*trnA*-*trnF*-*trnK*-*trnL2*-*trnQ*-*trnH*-*trnM3*
C2	7,011–16,819	*B. amorpha* strain GYU-BMZ01	*nad2*-*nad3*-*atp9*-*cox2*-*trnR1*-*nad4L*-*nad5*-*cob*
C3	16,818–21,192	*B. amorpha* strain GYU-BMZ01	*cob*-*trnC*-*cox1*
C4	21,302–24,514	*B. amorpha* strain GYU-BMZ01	*nad1*-*nad4*
C5	25,713–28,925	*B. amorpha* strain GYU-BMZ01	*nad4*-*atp8*-*atp6*-*rns*
C6	29,091–29,353	*B. amorpha* strain GYU-BMZ01	*trnD*-*trnS*-*trnN*
C7	29,342–30,157	*B. amorpha* strain GYU-BMZ01	*trnN*-*cox3*
C8	30,529–31,900	*B. amorpha* strain GYU-BMZ01	*nad6*-*trnV*
C9	31,954–32,191	*B. amorpha* strain GYU-BMZ01	*trnI*-*trnS*-*trnW*

**Figure 9:**
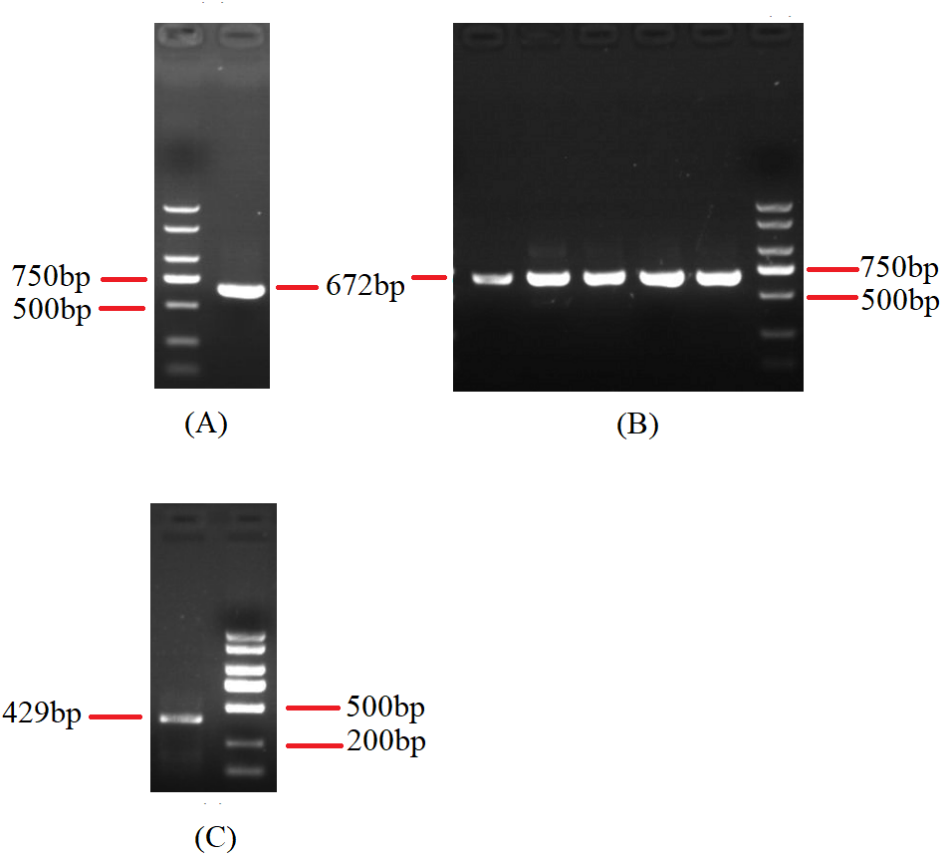
Agarose gel electropherogram of cDNA PCR product of *cox3*-*trnG*-*nad6* and *trnP*-*rnl*. (A) Agarose gel electropherogram of cDNA PCR product of *cox3*-*trnG*-*nad6* in mitogenome of *B. bassiana* strain GYU-BMZ04. (B) Agarose gel electropherogram of cDNA PCR product of *cox3*-*trnG*-*nad6* for five *Beauveria* strains, which were *B. amorpha* strain GYU-BMZ01, *B. caledonica* strain ATCC 64970, *B. brongniartii* strain GYU-BMZ03, *B. pseudobassiana* strain ATCC 90518, and *B. bassiana* strain YMM from left to right. (C) Agarose gel electropherogram of cDNA PCR product of *trnP*-*rnl* in mitogenome of *B. pseudobassiana* strain ATCC 90518. The markers were from the DL2000 Plus DNA Marker, which from top to bottom are 2,000, 1,500, 1,000, 750, 500, 250, 100 bp.

Polycistronic transcripts are subsequently processed into smaller mono-, di-, or tricistronic RNAs (Barth et al., 2001). *trnG* is 53 bp apart from *nad6*. For verification, *cox3*-*trnG*-*nad6* can be shown using cDNA and PCR in mitogenome of *B. bassiana* strain GYU-BMZ04 (Figs. 9A, 9B, Files S1 and S5). *trnP* is approximately 25 bp away from *rnl*. For verification, *trnP*-*rnl* can be shown using cDNA and PCR in mitogenome of *B. pseudobassiana* strain ATCC 90518 (Fig. 9C, Files S1 and S6). All the genes in the mitogenomes of the *Beauveria* species were located on the same strand. Since *Beauveria* species tends to produce relatively long polycistronic transcripts, we propose that only one polycistronic transcript (*trnM*_(1)_-*trnL*_(1)_-*trnA*-*trnF*-*trnK*-*trnL*_(2)_-*trnQ*-*trnH*-*trnM*_(2)_-*nad2*-*nad3*-*atp9*-*cox2*-*trnR*_(1)_-*nad4L*-*nad5*-*cob*-*trnC*-*cox1*-*trnR*_(2)_-*nad1*-*nad4*-*atp8*-*atp6*-*rns*-*trnY*-*trnD*-*trnS*-*trnN*-*cox3*-*trnG*-*nad6*-*trnV*-*trnI*-*trnS*-*trnW*-*trnP*-*rnl* (*rps3*)-*trnT*-*trnE*-*trnM*_(3)_) was processed from the mitogenome. For *B. bassiana* strain ARSEF 8028 (Fig. 7E and Table 2), *trnM*_(3)_ ended at base 5,543 and contig A ended at base 5,664 located in the control region. Therefore, transcription initiation sites may be located in the control region.

## Conclusions

In the present study, we utilized Illumina DNA and RNA sequencing data to assemble and annotate mitogenome of *Beauveria* species. The short RNA-seq reads from next-generation sequencing can improve the mitochondrial annotation accuracy and help study polycistronic transcripts and RNA processing. The pipeline of this study offers a reference for mitochondrial transcriptome. *Beauveria* species tend to process the growing polycistronic transcript first, and the poly A tail was added to the polycistronic transcript before splicing. The small proportion of mitochondrial transcripts among the total transcripts for *Beauveria* species, *B*. *bassiana* transcriptome sequenced by PacBio sequencing only have 11 full-length transcripts from mitogenome in previous study (Wang et al., 2020), the Oxford Nanopore Technology (ONT) may be more advantageous than PacBio technology. The presence of a largen of intron regions is interesting and further studies are needed to investigate the underlying mechanisms creating and preserving such RNA processing. Finally, the results of this study could be applied to other areas for fungal mitogenome.

##  Supplemental Information

10.7717/peerj.14067/supp-1 Table S1 Composition and skewness of mitogenomes of 8*Beauveria* Base composition, AT%, AT-skew and GC-skew mitogenomes of 8*Beauveria*.

10.7717/peerj.14067/supp-2 File S1 Peak figure of Sanger sequencing

10.7717/peerj.14067/supp-3 File S2 Target region, PCR primer information and PCR product sequences of *cox2* of *B*. *bassiana* strain GYU-BMZ04

10.7717/peerj.14067/supp-4 File S3 Target region, PCR primer information and PCR product sequences of *rns*-*trnY*-*trnD*-*trnS*-*trnN*-*cox3* region of *B*. *bassiana* strain GYU-BMZ04

10.7717/peerj.14067/supp-5 File S4 Target region, PCR primer information and PCR product sequences of *cox1*-*trnR*-*nad1* region of *B*. *bassiana* strain GYU-BMZ04

10.7717/peerj.14067/supp-6 File S5 Target region, PCR primer information and PCR product sequences of *cox3*-*trnG*-*nad6* region of *B*. *bassiana* strain GYU-BMZ04

10.7717/peerj.14067/supp-7 File S6 Target region, PCR primer information and PCR product sequences of *trnP*-*rnl* region of *B*.* pseudobassiana* strain ATCC 90518

## Additional Information and Declarations

### DNA Deposition

The following information was supplied regarding the deposition of DNA sequences: The data is available at GenBank: OM782663–OM782670.

### Data Availability

The following information was supplied regarding data availability: The data is available at BioProject: PRJNA809086; SAMN26143421– SAMN26143427, and SRR18094432– SRR18094445.
