# Supplementary material for: Comparative mitogenome analysis reveals mitochondrial genome characteristics in eight strains of Beauveria
Source: PeerJ. 2022 Sep 28;10:e14067. doi: 10.7717/peerj.14067 (PMC9526403; doi:10.7717/peerj.14067)
Supplement: Table S1 — Base composition, AT%, AT-skew and GC-skew mitogenomes of 8Beauveria. [file peerj-10-14067-s001.docx]

**Table S1. Composition and skewness of mitogenomes of 8 *Beauveria*.**

| **Species** | **Size (bp)** | **A (num)** | **T (num)** | **G (num)** | **C (num)** | **AT%** | **GC%** | **AT-skew** | **GC-skew** |
| --- | --- | --- | --- | --- | --- | --- | --- | --- | --- |
| *B. amorpha* strain GYU-BMZ01 | 32316 | 11865 | 11743 | 4821 | 3887 | 73.05 | 26.95 | 0.0052 | 0.1073 |
| *B. amorpha* strain GYU-BMZ02 | 32316 | 11865 | 11743 | 4821 | 3887 | 73.05 | 26.95 | 0.0052 | 0.1073 |
| *B. bassiana* strain YMM | 28815 | 10424 | 10584 | 4319 | 3488 | 72.91 | 27.09 | -0.0076 | 0.1064 |
| *B. bassiana* strain GYU-BMZ04 | 29968 | 10864 | 10946 | 4526 | 3632 | 72.78 | 27.22 | 0.0052 | 0.1073 |
| *B. bassiana* strain ARSEF 8028 | 31240 | 11410 | 11371 | 4684 | 3775 | 72.92 | 27.08 | 0.0017 | 0.1075 |
| *B. brongniartii* strain GYU-BMZ03 | 35999 | 13298 | 12972 | 5400 | 4329 | 72.97 | 27.03 | 0.0124 | 0.1101 |
| *B. caledonica* strain ATCC 64970 | 30223 | 10991 | 11065 | 4527 | 3640 | 72.98 | 27.02 | -0.0034 | 0.1086 |
| *B. pseudobassiana* strain ATCC 90518 | 26850 | 9545 | 9914 | 4100 | 3291 | 72.47 | 27.53 | -0.0190 | 0.1095 |
