# Supplementary material for: Comparative mitogenome analysis reveals mitochondrial genome characteristics in eight strains of Beauveria
Source: PeerJ. 2022 Sep 28;10:e14067. doi: 10.7717/peerj.14067 (PMC9526403; doi:10.7717/peerj.14067)
Supplement: File S2 [file peerj-10-14067-s003.docx]

**1. Target region:**

>cox2 B. bassiana strain GYU-BMZ04

TCAGCATATTAGTTGGTGTTAATTACTAATTAGTTAATAGGGATGGTGGTGGGTACGTATAAGCGTAATTAAAAATTTTTTATTTAGAAATGGATATTTAAAATAAAATGACAAATATGTTAAATTCAATTATTAGCTTTGATGCTCCTGAAGCATGAGGTATTTATTTTCAAGATAGTGCTACTCCACAAATGGAAGGATTAATAGAATTACATGATAATATTATGTATTATTTAGTTTTAATATTATTTGCTGTAGGATGAGTATTATTTTCAATAGTAAAAAATTTTGCTATGAAAAATTCACCTATATCACATAAATATTTAAATCATGGTAGAAGTGTGCCTTCTCAAAAGTGTTTTAATTTTAATATTCAGAGTATAAAGTTTTACAGTACTTCTTCTGTTAACAGTATAAAGTTTTATGAAGATGCTTTCTCTATGAGAAAATTAATTATAAAAGAGAATAAAAATAAATCAGGGATTTATAAATGAACCAATAAAGTAACGAATGATATATATATTGGTCAATCTATAGATTTAGCAAAAAGATTTATTAGATATTTTAATCTTAGTTATTTAAAAAATAGAGAAAGCCTTGTAATAAGTAGAGCTTTAATTAAATATGGTTATTCTAATTTTTCATTAGATATATTAGAATATTGTGATATTAAAGATTTAACAGAAAGAGAACAATATTATATGGATAAATTAAATCCTAAATATAATACTTTAAAAATAGCAGGTAGTTCTTCAGGTCATAAACTTTCTGAAGAAACTAAAGCACTTATGTCTTTAACAAGAGCTACTATAAATAATTTAGGTAAAATTCATACTGAAGAAACTAAAGAATTAATGAGACAAAAAGCTTTAGGTAGAAAACATTCTGCCCTTCGGCCTAGGCCCCTGGACCTAGTCCTGGGCCGCGAGGAAGAAACTTTATTAAAGATGAGTATTGCCAGAGGTTATTCTGTAGATATCTTAGAAAAATGTGATTCAGAAGGCTTTAAATTAATAGGTAGTTTTGTTTCGATAAGAAAAGCTGCTAAATTTTTAGAAATTAGTGCTAATACTGTAAAACTTTATATAAACTCAGGTAAAATATTTAAAAATAGATATAAATTCGTAAGTATTAAATAGAATTAAAAAAACTAAGAGAAAAATTCCACTATATGCTGGAAACTCCTAAAGCCTTTAGGTACTATAAAGATAATAATATATAAACTTTATGAGTGATAACCCTAAAGGATGTACAATGGATTATCAGCAGGAAACCAAAATAAGTAGGATCCTCAGAGACTAAACGTGGAAACTTTATAGATATAAAGTAAGATATAGTCCGGTTAAGTATGAAAGTGCTTAAGTTTAATCGACATTGATTGAATTAATATGAACTATTACACCAGCATTAGTTTTAATATTAATCGCTTTCCCTTCATTTAAATTATTATATTTAATGGATGAAGTGAATGATCCATCATTAACTATTATAGCAGAAGGTCATCAATGATATTGAAGTTATCAATATCCTGATTTTATAAATTCAGATGGTGAATTTATAGAATTTGATTCTTATATTGTACCAGATTCAGATTTAGAAGACGGTGGGTTAAGAATGTTAGAAGTGGATAATAGAGTTATGTTACCTGAGTTAACTCATACAAGATTAGTAGCTACTAGTGGTGATGTTATACACTCATTAGCTTGTCCAGCTTTAGGTATTAAATGTGATGCATATCCAGGTAGATTAAATCAATTATCAATATTTGTTAATAGACCAGGTGTATTTTATGGTCAATGTTCTGAAATATGCGGAATATTACATAGCTCAATGCCTCTTGTATTCCAATCTACAGATTTACCAACATTTTTAAATTGATTATATAATGCATAATTGTAAATATACATACATATAGTAATTTCAC

note: Nucleotides of intron were highlighted in green colour. Nucleotides of start codon and stop codon were showed in red colour. Nucleotides of the cDNA primers were underlines and highlighted in yellow colour. Nucleotides of the DNA primers were underlines and highlighted in blue colour.

**2. cDNA PCR amplification**

size：888bp

Primer:

cox2_cDNA_F: TCAGCATATTAGTTGGTG

cox2_cDNA_R: GTGAAATTACTATATGTATG

**sequencing result：**

>cox2_cDNA

GGTGGTGGGTACGTATAAGCGTAATTAAAAATTTTTTATTTAGAAATGGATATTTAAAATAAAATGACAAATATGTTAAATTCAATTATTAGCTTTGATGCTCCTGAAGCATGAGGTATTTATTTTCAAGATAGTGCTACTCCACAAATGGAAGGATTAATAGAATTACATGATAATATTATGTATTATTTAGTTTTAATATTATTTGCTGTAGGATGAGTATTATTTTCAATAGTAAAAAATTTTGCTATGAAAAATTCACCTATATCACATAAATATTTAAATCATGGTACATTGATTGAATTAATATGAACTATTACACCAGCATTAGTTTTAATATTAATCGCTTTCCCTTCATTTAAATTATTATATTTAATGGATGAAGTGAATGATCCATCATTAACTATTATAGCAGAAGGTCATCAATGATATTGAAGTTATCAATATCCTGATTTTATAAATTCAGATGGTGAATTTATAGAATTTGATTCTTATATTGTACCAGATTCAGATTTAGAAGACGGTGGGTTAAGAATGTTAGAAGTGGATAATAGAGTTATGTTACCTGAGTTAACTCATACAAGATTAGTAGCTACTAGTGGTGATGTTATACACTCATTAGCTTGTCCAGCTTTAGGTATTAAATGTGATGCATATCCAGGTAGATTAAATCAATTATCAATATTTGTTAATAGACCAGGTGTATTTTATGGTCAATGTTCTGAAATATGCGGAATATTACATAGCTCAATGCCTCTTGTATTCCAATCTACAGATTTACCAACATTTTTAAATTGATTATATAATGCATAATTGTAAATAT

**3. mito PCR amplification**

size：1305bp

Primer:

cox2_mito_F:GATAGTGCTACTCCACAAATGG

cox2_mito_R:GATGGATCATTCACTTCATC

**sequencing result**

>cox2_mito_1, cox2_mito_2

TATTATGTATTATTTAGTTTTAATATTATTTGCTGTAGGATGAGTATTATTTTCAATAGTAAAAAATTTTGCTATGAAAAATTCACCTATATCACATAAATATTTAAATCATGGTAGAAGTGTGCCTTCTCAAAAGTGTTTTAATTTTAATATTCAGAGTATAAAGTTTTACAGTACTTCTTCTGTTAACAGTATAAAGTTTTATGAAGATGCTTTCTCTATGAGAAAATTAATTATAAAAGAGAATAAAAATAAATCAGGGATTTATAAATGAACCAATAAAGTAACGAATGATATATATATTGGTCAATCTATAGATTTAGCAAAAAGATTTATTAGATATTTTAATCTTAGTTATTTAAAAAATAGAGAAAGCCTTGTAATAAGTAGAGCTTTAATTAAATATGGTTATTCTAATTTTTCATTAGATATATTAGAATATTGTGATATTAAAGATTTAACAGAAAGAGAACAATATTATATGGATAAATTAAATCCTAAATATAATACTTTAAAAATAGCAGGTAGTTCTTCAGGTCATAAACTTTCTGAAGAAACTAAAGCACTTATGTCTTTAACAAGAGCTACTATAAATAATTTAGGTAAAATTCATACTGAAGAAACTAAAGAATTAATGAGACAAAAAGCTTTAGGTAGAAAACATTCTGCCCTTCGGCCTAGGCCCCTGGACCTAGTCCTGGGCCGCGAGGAAGAAACTTTATTAAAGATGAGTATTGCCAGAGGTTATTCTGTAGATATCTTAGAAAAATGTGATTCAGAAGGCTTTAAATTAATAGGTAGTTTTGTTTCGATAAGAAAAGCTGCTAAATTTTTAGAAATTAGTGCTAATACTGTAAAACTTTATATAAACTCAGGTAAAATATTTAAAAATAGATATAAATTCGTAAGTATTAAATAGAATTAAAAAAACTAAGAGAAAAATTCCACTATATGCTGGAAACTCCTAAAGCCTTTAGGTACTATAAAGATAATAATATATAAACTTTATGAGTGATAACCCTAAAGGATGTACAATGGATTATCAGCAGGAAACCAAAATAAGTAGGATCCTCAGAGACTAAACGTGGAAACTTTATAGATATAAAGTAAGATATAGTCCGGTTAAGTATGAAAGTGCTTAAGTTTAATCGACATTGATTGAATTAATATGAACTATTACACCAGCATTAGTTTTAATATTAATCGCTT
