# Supplementary material for: Comparative mitogenome analysis reveals mitochondrial genome characteristics in eight strains of Beauveria
Source: PeerJ. 2022 Sep 28;10:e14067. doi: 10.7717/peerj.14067 (PMC9526403; doi:10.7717/peerj.14067)
Supplement: File S3 [file peerj-10-14067-s004.docx]

**1. Target region:**

>rns_trnY_trnD_trnS_trnN_cox3 B. bassiana strain GYU-BMZ04

GGAGTTTATACAATAAGTTTGCTCTTTTATAGTAAAAAAATAAAATAATTAGATTTTGTATATATTATTATAAAATTCTTCGCATGTATAGCTCTAATTAGTGTTAAGTCGAAATACGGTTCGCGTAGTGGAAGTTGCGCGGGAATAATTGATCCTGAACAATAGATAAGAGAGTTAGCTTAATGCTACTCTTAAGGAGGGTTCCTTTATTGGCAAGAAGGGCTAAACTGTAAATTTAGTACATTATAATGTTTTGAGGGTTCGAATCCCTCGTCTCCTATTAGATCTAGTAACTTAATAGGTAAAGGATTTCCTTGTCACGGAAATAGATGTCGGTTCGATGCTGATCTAGGTCGAGATTAATTTAAAGCACTAGCTTGCGCTATTTTTTTTAACAAAAAAATATAAAGCAGTGATTTAGCTTTAACTCACAGGAAAAATCTCCATTGGTAGGGTAAGACACTTGCTATGTGTTATGTTTTTACATTTAGGTGTTCGATTCACCTTTTTTCCGTCAAAGTTCTTATAGCTCAACGGTAGAGCATAATACTGTTAATATTATGATAAATGTTCGATTCATTTTAAGGACTCATATATAAATAAAGAAATCTTTATTCAGTAAACTTAAGCTAGAAAATTAATAACAAAAAACATGACAAATTTAACAAGAAATCATTTTCAAGATCATCCTTTTCATTTAGTATCTCCTAGTCCTTGACCTTTATATACAAGTATATCATTGTTTAC

note: Nucleotides of *rns* were highlighted in yellow colour. Nucleotides of *trnY* were highlighted in green colour. Nucleotides of *trnS* were highlighted in purple colour. Nucleotides of *trnN* were highlighted in blue colour. Nucleotides of start codon of *cox3* were showed in red colour. Nucleotides of the cDNA primers were underlines.

**2. cDNA PCR amplification**

size：747bp

Primer:

rns_trnY_trnD_trnS_trnN_cox3_F: GGAGTTTATACAATAAGTTTGC

rns_trnY_trnD_trnS_trnN_cox3_R: GTAAACAATGATATACTTGT

**sequencing result：**

>rns_trnY_trnD_trnS_trnN_cox3_1

AGATTTTGTATATATTATTATAAAATTCTTCGCATGTATAGCTCTAATTAGTGTTAAGTCGAAATACGGTTCGCGTAGTGGAAGTTGCGCGGGAATAATTGATCCTGAACAATAGATAAGAGAGTTAGCTTAATGCTACTCTTAAGGAGGGTTCCTTTATTGGCAAGAAGGGCTAAACTGTAAATTTAGTACATTATAATGTTTTGAGGGTTCGAATCCCTCGTCTCCTATTAGATCTAGTAACTTAATAGGTAAAGGATTTCCTTGTCACGGAAATAGATGTCGGTTCGATGCTGATCTAGGTCGAGATTAATTTAAAGCACTAGCTTGCGCTATTTTTTTTAACAAAAAAATATAAAGCAGTGATTTAGCTTTAACTCACAGGAAAAATCTCCATTGGTAGGGTAAGACACTTGCTATGTGTTATGTTTTTACATTTAGGTGTTCGATTCACCTTTTTTCCGTCAAAGTTCTTATAGCTCAACGGTAGAGCATAATACTGGTAATATTATGATAAATGGTCGATTCATTTTAAGGACTCATATATAAATAAAGAAATCTTTATTCAGTAAACTTAAGCTAGAAAATTAATAACAAAAAACATGACAAATTTAACAAGAAATCATTTTCAAGATCATCCTTTTCATTTAGTATCTCCTAGTCCTTGACCTTTATATACA
