# Supplementary material for: Comparative mitogenome analysis reveals mitochondrial genome characteristics in eight strains of Beauveria
Source: PeerJ. 2022 Sep 28;10:e14067. doi: 10.7717/peerj.14067 (PMC9526403; doi:10.7717/peerj.14067)
Supplement: File S4 [file peerj-10-14067-s005.docx]

**1. Target region:**

>cox1_trnR_nad1 B. bassiana strain GYU-BMZ04

GTTGCAAGTAGAAATCCTTGATTAATACCTGGATTCTATACAGATATATTACAAAGTAATTTAAATAGATCATACAGTAGTTTAGAATGAGGATTATCAAGTCCACCTAAACCTCATGCATTTGTAAGTTTACCTTTACAATCTAGTCTAGGGAAGAAACACATTTAATAGCTTGCACACAAATTGATTACACAAGATATGAACATTTATACTTATATTTTTCAAGAAAAATTATTGAATAAAACTAATACTAATAATAGTAATATTATTAGTAATGTCTTATTCGATATTAGATGTTCCTACAACATATTTTGCGGCACCTAATCCAAAATACTCCTCTGACTTTTGAACTACTGTTTTAGAGAATAAAGAAAATATTTTTAATTCTTCTGGGGATAATAATACCCTTAATGTTGAATCTAGACCAACTACCCCTATGGTTGAACCTAGACCAACTATTCCTGTGGTTGAACCTATTCCTGTAATTGAACCTAGGCCAACTATCCCTGTGATTGAACCTATTCCTGTGGTTGAACCTAGACCAACTATTCCTATGGTTGAACATAGACCAGAAGTTTATACTGTTGAACCTAGACCATCTAGTTCTCAAGTTAACACTCATAGAGCTATGGATATTAACAATTTTATATGACGTGAGGATATTCATGGTTATAAATACCCCTCTCAGGGTTTACAAACAAATCCTTCAATTAATTCACATATTAACTACAATGTAAGTAATACTATGAATTCTAGCAGTTACACTTTCCAAAGTAATTTAAATCCTACATATACTAATTATACTGTTGGAAATCTAAGTAATTTAAATCCTACATATACTAATTATACTGTTGGAAATCAAAGTAATTTAAATCCTACATATGTTAATTATACTGTTGGAAATCAAAGTACTATTATGCAAAATGTACCTGAATCTAGTTTTCAAGCTCAAAGTACTATTATGCAAAATGTAGCTGAATCTAGTAATATGCCTGAAATAAGAGCTATGGCTGAATCTAGTTTTCAAGCTCAAAGTAATATATCTGAAATGAGGGTTATAGCTGAATCCAGTTCTCAAGCTCAAAGTAATATATCTGAAATGAGGGTTATAACTGAATCTGGTTTTACTAAACCAAATTTAAAACCTATATGAATTGAAAATTATAGTAATGTAGTAGATACTGCTACATCTAAATGTTTTAATCCCATGTTTTCAAGTTTACTTCCTTGACAGGAAATAGAACTTTCTGAGATAACTAAATTAAATAGACCTAAAATAGTAGGTATTAATTTTGATTTTTCTCATCAAAATATAGAAGCAGTACTTCAAGAATTTATGTTACCAAAGGCATTTTATGTGTCTCATGAACATGTTCCTGGTTTAACTCCAAGTAATTATAAAGAATATATTATTAATTACTATCATACTAAGGCCTGTAATAAACCTTCTATTTTACTTCAGGCAGTTTTGATCGAAAATGGAGATGTTTTGAAAGTAATTAAAGGTAGATAGTATAAGTATAATTATACTTCTAATCTCATTAGCTCAATGGTAGAGCATAATACTTCTAATATTACAATCCTAGTTCGATTCTAGGATGAGATTATTATTTTAAGACTTAGCTTGCTTAATAAGATAAACAAAATATGGAAATAGATTAAACTATTTTATTTCTATTAATATCTTATTATTTATAGGCTATTATATTAACAAAATAGATTATAGGATACACCTATAAATTAAAACAGCTTCGCAAAATGATTATTTCAAGGCTATAAAATAAAGCTCGTTACTTATAGTTTCTTTAGCTTTATATATGCTAGCATATCATACTAATAGTAGAGCAAAGAGTTTATAATAAAGATTGCTATATGTATACATCAATATATTATATAGTAAGGCTATGAAATAAAGCTCTTTATTATTATTACTATAACTAAAGCTTTTAACAAATTAATTTAATACCTACCATACATATTAATAAATAAACAATGTTAATATTATGATTAGTCGCTGCTAATGCGAAGCCGCACTATTTTTTTAATATAAAACTATAGAATTATTTTTTTTTATAATTATTTATTAAGTGTAACTATGTAAAAACAATGAATATAACTATTTTGTCCATAATAGAAACTATTATTTTAATGCTTCCTGCATTATTAGTAGTAGCTTATGTAACAGTAGCTGAAAGAAAAACAATGGCCAGTATGCAAAGAAGATTAGGGCCTAATGCTGTAGGTTATTATGGACTATTACAAGCTTTTGC

note: Nucleotides of *trnR*_(2)_ were highlighted in green colour. Nucleotides of stop codon of *cox1* and start codon of *nad6* were showed in red colour. Nucleotides of the cDNA primers were underlines and highlighted in yellow colour.

**2. cDNA PCR amplification**

size：2270bp

Primer:

cox1_trnR_nad1_F: GTTGCAAGTAGAAATCCTTG

cox1_trnR_nad1_R: GCAAAAGCTTGTAATAGTCC

**sequencing result：**

>cox1_trnR_nad1_1, cox1_trnR_nad1_2, cox1_trnR_nad1_3

GTAATTTAAATAGATCATACAGTAGTTTAGAATGAGGATTATCAAGTCCACCTAAACCTCATGCATTTGTAAGTTTACCTTTACAATCTAGTCTAGGGAAGAAACACATTTAATAGCTTGCACACAAATTGATTACACAAGATATGAACATTTATACTTATATTTTTCAAGAAAAATTATTGAATAAAACTAATACTAATAATAGTAATATTATTAGTAATGTCTTATTCGATATTAGATGTTCCTACAACATATTTTGCGGCACCTAATCCAAAATACTCCTCTGACTTTTGAACTACTGTTTTAGAGAATAAAGAAAATATTTTTAATTCTTCTGGGGATAATAATACCCTTAATGTTGAATCTAGACCAACTACCCCTATGGTTGAACCTAGACCAACTATTCCTGTGGTTGAACCTATTCCTGTAATTGAACCTAGGCCAACTATCCCTGTGATTGAACCTATTCCTGTGGTTGAACCTAGACCAACTATTCCTATGGTTGAACATAGACCAGAAGTTTATACTGTTGAACCTAGACCATCTAGTTCTCAAGTTAACACTCATAGAGCTATGGATATTAACAATTTTATATGACGTGAGGATATTCATGGTTATAAATACCCCTCTCAGGGTTTACAAACAAATCCTTCAATTAATTCACATATTAACTACAATGTAAGTAATACTATGAATTCTAGCAGTTACACTTTCCAAAGTAATTTAAATCCTACATATACTAATTATACTGTTGGAAATCTAAGTAATTTAAATCCTACATATACTAATTATACTGTTGGAAATCAAAGTAATTTAAATCCTACATATGTTAATTATACTGTTGGAAATCAAAGTACTATTATGCAAAATGTACCTGAATCTAGTTTTCAAGCTCAAAGTACTATTATGCAAAATGTAGCTGAATCTAGTAATATGCCTGAAATAAGAGCTATGGCTGAATCTAGTTTTCAAGCTCAAAGTAATATATCTGAAATGAGGGTTATAGCTGAATCCAGTTCTCAAGCTCAAAGTAATATATCTGAAATGAGGGTTATAACTGAATCTGGTTTTACTAAACCAAATTTAAAACCTATATGAATTGAAAATTATAGTAATGTAGTAGATACTGCTACATCTAAATGTTTTAATCCCATGTTTTCAAGTTTACTTCCTTGACAGGAAATAGAACTTTCTGAGATAACTAAATTAAATAGACCTAAAATAGTAGGTATTAATTTTGATTTTTCTCATCAAAATATAGAAGCAGTACTTCAAGAATTTATGTTACCAAAGGCATTTTATGTGTCTCATGAACATGTTCCTGGTTTAACTCCAAGTAATTATAAAGAATATATTATTAATTACTATCATACTAAGGCCTGTAATAAACCTTCTATTTTACTTCAGGCAGTTTTGATCGAAAATGGAGATGTTTTGAAAGTAATTAAAGGTAGATAGTATAAGTATAATTATACTTCTAATCTCATTAGCTCAATGGTAGAGCATAATACTTCTAATATTACAATCCTAGTTCGATTCTAGGATGAGATTATTATTTTAAGACTTAGCTTGCTTAATAAGATAAACAAAATATGGAAATAGATTAAACTATTTTATTTCTATTAATATCTTATTATTTATAGGCTATTATATTAACAAAATAGATTATAGGATACACCTATAAATTAAAACAGCTTCGCAAAATGATTATTTCAAGGCTATAAAATAAAGCTCGTTACTTATAGTTTCTTTAGCTTTATATATGCTAGCATATCATACTAATAGTAGAGCAAAGAGTTTATAATAAAGATTGCTATATGTATACATCAATATATTATATAGTAAGGCTATGAAATAAAGCTCTTTATTATTATTACTATAACTAAAGCTTTTAACAAATTAATTTAATACCTACCATACATATTAATAAATAAACAATGTTAATATTATGATTAGTCGCTGCTAATGCGAAGCCGCACTATTTTTTTAATATAAAACTATAGAATTATTTTTTTTTATAATTATTTATTAAGTGTAACTATGTAAAAACAATGAATATAACTATTTTGTCCATAATAGAAACTATTATTTTAATGCTTCCTGCATTATTAGTAGTAGCTTATGTAACAGTAGCTGAAAGAAAAACAATGGCCAGTATGCAAAGAAGATTAG
