# Supplementary material for: Comparative mitogenome analysis reveals mitochondrial genome characteristics in eight strains of Beauveria
Source: PeerJ. 2022 Sep 28;10:e14067. doi: 10.7717/peerj.14067 (PMC9526403; doi:10.7717/peerj.14067)
Supplement: File S5 [file peerj-10-14067-s006.docx]

**1. Target region:**

>cox3_trnG_nad6 B. bassiana strain GYU-BMZ04

GTGTATTTGGTACATGTTTCTTTTTTGGAACAGGGTTCCATGGATTACATGTTATAATAGGTACTATATTTTTAGCAGTAGGTCTATGAAGAATTATATCATATCATTTAACTGATCATCACCATTTAGGTTATGAAGCAGGTATATTATACTGACATTTTGTGGATGTAGTTTGATTATTCTTATATGTATCAATGTATTATTGAGGTTCTTAATATAGAAACTTAAGATATATATCTTAATAATAATTATTAAGATAAGCGGGTATAGGTTAAAGGTAGACTTTTCGCTTTCCACCCGAAATGTGTCAGTTCGATTCTGACTATCCGTATATTTAATATTATAATATTATATTATATCTTTTTAACCAGTTATAAATTAACTATGATTTGATCTATACATGAATATTATTTTTCAGGTTATACTGTAGAATTCTTAGATATATTAAGTGTTATAGCAGTATTATTTGGTATAACAGTAATTATAAATAAAAACCCTATAGGTTCTTTATTATTTTTAATAGGGTTATTTGCTTCAATTTCAGTCTATTTAATATTATCAGGATTAACATTTATTGGTTTTTCTTATTTAATAGTATATATAGGAGCAGTATCTATTTTATTTTTATTTATATTAATGCTTATTAATATAAGAACAAGTGAATTACAAAGT

note: Nucleotides of *trnG* were highlighted in green colour. Nucleotides of stop codon of *cox3* and start codon of *nad6* were showed in red colour. Nucleotides of the cDNA primers were underlines and highlighted in yellow colour.

**2. cDNA PCR amplification**

size：672bp

Primer:

cox3_trnG_nad6_F: GTGTATTTGGTACATGTTTC

cox3_trnG_nad6_R: ACTTTGTAATTCACTTGTTC

**sequencing result：**

>cox3_trnG_nad6_1

GTTATAATAGGTACTATATTTTTAGCAGTAGGTCTATGAAGAATTATATCATATCATTTAACTGATCATCACCATTTAGGTTATGAAGCAGGTATATTATACTGACATTTTGTGGATGTAGTTTGATTATTCTTATATGTATCAATGTATTATTGAGGTTCTTAATATAGAAACTTAAGATATATATCTTAATAATAATTATTAAGATAAGCGGGTATAGGTTAAAGGTAGACTTTTCGCTTTCCACCCGAAATGTGTCAGTTCGATTCTGACTATCCGTATATTTAATATTATAATATTATATTATATCTTTTTAACCAGTTATAAATTAACTATGATTTGATCTATACATGAATATTATTTTTCAGGTTATACTGTAGAATTCTTAGATATATTAAGTGTTATAGCAGTATTATTTGGTATAACAGTAATTATAAATAAAAACCCTATAGGTTCTTTATTATTTTTAATAGGGTTATTTGCTTCAATTTCAGTCTATTTAATATTATCAGGATTAACATTTATTGGTTTTTCTTATTTAATAGTATATATAGGAGCAGTATCTATTTTATTTTTATTTATATTAATGCTTATTAATATAAGAACA
