# Supplementary material for: Comparative mitogenome analysis reveals mitochondrial genome characteristics in eight strains of Beauveria
Source: PeerJ. 2022 Sep 28;10:e14067. doi: 10.7717/peerj.14067 (PMC9526403; doi:10.7717/peerj.14067)
Supplement: File S6 [file peerj-10-14067-s007.docx]

**1. Target region:**

>trnP_rnl B. pseudobassiana strain ATCC 90518

TCGGATATTCTCTGAAAGAGTATAGTTTAATAGGCAAAATACGAAGCTTCAACCTTCAGGTTCTTGGTTCAAATCCAAGTACTCTTGTATGTATAATAAATTCGGATTAGGGCCGGGCGGTCAGGCACTTCGTTTGGGACGGAGATTAGTTATGTTCGATTCATAATAATCCGAATATTAGAACATTTAGAAATAAAAATAAGATATATAAATTATACTTATTAGCTTGTTTAACAAGTAATGTAAGTATGTAAAGAAATTATTATGCAATTCTAGCTATGTATTAAGGAGAGCAAATTTTATATAAAAAGGAATATTATATATTCCTAGTTAATAAGCTCTAGAATAAATTTAATAATAAACGAAGTGAAGTGAAATATCTCAGTAACTTCAGGAAAAGAAATCAAAAGAGATTCTATGAATAGCGTG

note: Nucleotides of *rns* were highlighted in yellow colour. Nucleotides of *trnP* were highlighted in green colour. Nucleotides of the cDNA primers were underlines.

**2. cDNA PCR amplification**

size：429 bp

Primer:

trnP_rnl_F: TCGGATATTCTCTGAAAGAG

trnP_rnl_R: CACGCTATTCATAGAATCTC

**sequencing result：**

>trnP_rnl_1

CCTTCAGGTTCTTGGTTCAAATCCAAGTACTCTTGTATGTATAATAAATTCGGATTAGGGCCGGGCGGTCAGGCACTTCGTTTGGGACGGAGATTAGTTATGTTCGATTCATAATAATCCGAATATTAGAACATTTAGAAATAAAAATAAGATATATAAATTATACTTATTAGCTTGTTTAACAAGTAATGTAAGTATGTAAAGAAATTATTATGCAATTCTAGCTATGTATTAAGGAGAGCAAATTTTATATAAAAAGGAATATTATATATTCCTAGTTAATAAGCTCTAGAATAAATTTAATAATAAACGAAGTGAAGTGAAATATCTCAGTAACTTCAGGAAAAGAAATCAAAAGAGATTCT
